# Identification and combinatorial engineering of indole-3-acetic acid synthetic pathways in *Paenibacillus polymyxa*

**DOI:** 10.1186/s13068-022-02181-3

**Published:** 2022-08-11

**Authors:** Huimin Sun, Jikun Zhang, Wenteng Liu, Wenhui E, Xin Wang, Hui Li, Yanru Cui, Dongying Zhao, Kai Liu, Binghai Du, Yanqin Ding, Chengqiang Wang

**Affiliations:** College of Life Sciences, Shandong Key Laboratory of Agricultural Microbiology, State Key Laboratory of Crop Biology, Shandong Engineering Research Center of Plant-Microbia Restoration for Saline-Alkali Land, Shandong Agricultural University, 61 Daizong Street, Tai’an, 271018 China

**Keywords:** *Paenibacillus polymyxa*, PGPR, IAA, Metabolic pathway, Promoter engineering

## Abstract

**Background:**

*Paenibacillus polymyxa* is a typical plant growth-promoting rhizobacterium (PGPR), and synthesis of indole-3-acetic acid (IAA) is one of the reasons for its growth-promoting capacity. The synthetic pathways of IAA in *P. polymyxa* must be identified and modified.

**Results:**

*P. polymyxa* SC2 and its spontaneous mutant SC2-M1 could promote plant growth by directly secreting IAA. Through metabonomic and genomic analysis, the genes *patA*, *ilvB3*, and *fusE* in the native IPyA pathway of IAA synthesis in strain SC2-M1 were predicted. A novel strong promoter *P*_*04420*_ was rationally selected, synthetically analyzed, and then evaluated on its ability to express IAA synthetic genes*.* Co-expression of three genes, *patA*, *ilvB3*, and *fusE*, increased IAA yield by 60% in strain SC2-M1. Furthermore, the heterogeneous gene *iaam* of the IAM pathway and two heterogeneous IPyA pathways of IAA synthesis were selected to improve the IAA yield of strain SC2-M1*.* The genes *ELJP6_14505*, *ipdC,* and *ELJP6_00725* of the entire IPyA pathway from *Enterobacter ludwigii* JP6 were expressed well by promoter *P*_*04420*_ in strain SC2-M1 and increased IAA yield in the engineered strain SC2-M1 from 13 to 31 μg/mL, which was an increase of 138%.

**Conclusions:**

The results of our study help reveal and enhance the IAA synthesis pathways of *P. polymyxa* and its future application.

**Supplementary Information:**

The online version contains supplementary material available at 10.1186/s13068-022-02181-3.

## Highlights

1. Verifying an entire native IPyA pathway of IAA synthesis in *P. polymyxa.*
2. Introducing heterologous IAM and IPyA pathways of IAA synthesis to *P. polymyxa.*
3. Selecting and analyzing a novel strong promoter *P*_*04420*_ to express IAA synthesis genes.

**Supplementary Information:**

The online version contains supplementary material available at 10.1186/s13068-022-02181-3.

## Background

Many rhizobacteria play growth promotion and biological control functions for plants and are called plant growth-promoting rhizobacteria (PGPR) [1–3]. *Paenibacillus polymyxa* is a typical PGPR that can promote the growth, development, and stress resistance of plants [4–6]. *P. polymyxa*, formerly named *Bacillus polymyxa*, was reclassified to the genus *Paenibacillus* by Ash [7]. *P. polymyxa* is now widely separated from the rhizosphere soil of many plants, including crops such as tomatoes [8], Sudan grass [9], rice [10], cucumber [11], bean [12], sunflower [13], wheat [14], Arabidopsis [15], and *Lilium lancifolium* [5]. The growth-promoting mechanisms of *P. polymyxa* are diverse [16]. *P. polymyxa* can indirectly promote plant growth by improving the induced resistance of plants [17] and providing antagonistic properties to plant pathogens [18]. Furthermore, *P. polymyxa* can directly promote plant growth by fixing nitrogen [19], dissolving phosphorus [20], dissolving potassium [9], producing siderophores [21], secreting chitinase and volatile gases [22, 23], and enhancing the synthesis of ethylene [24], cytokinins [25], and indole-3-acetic acid (IAA) [26–28].

**Fig. 1:**
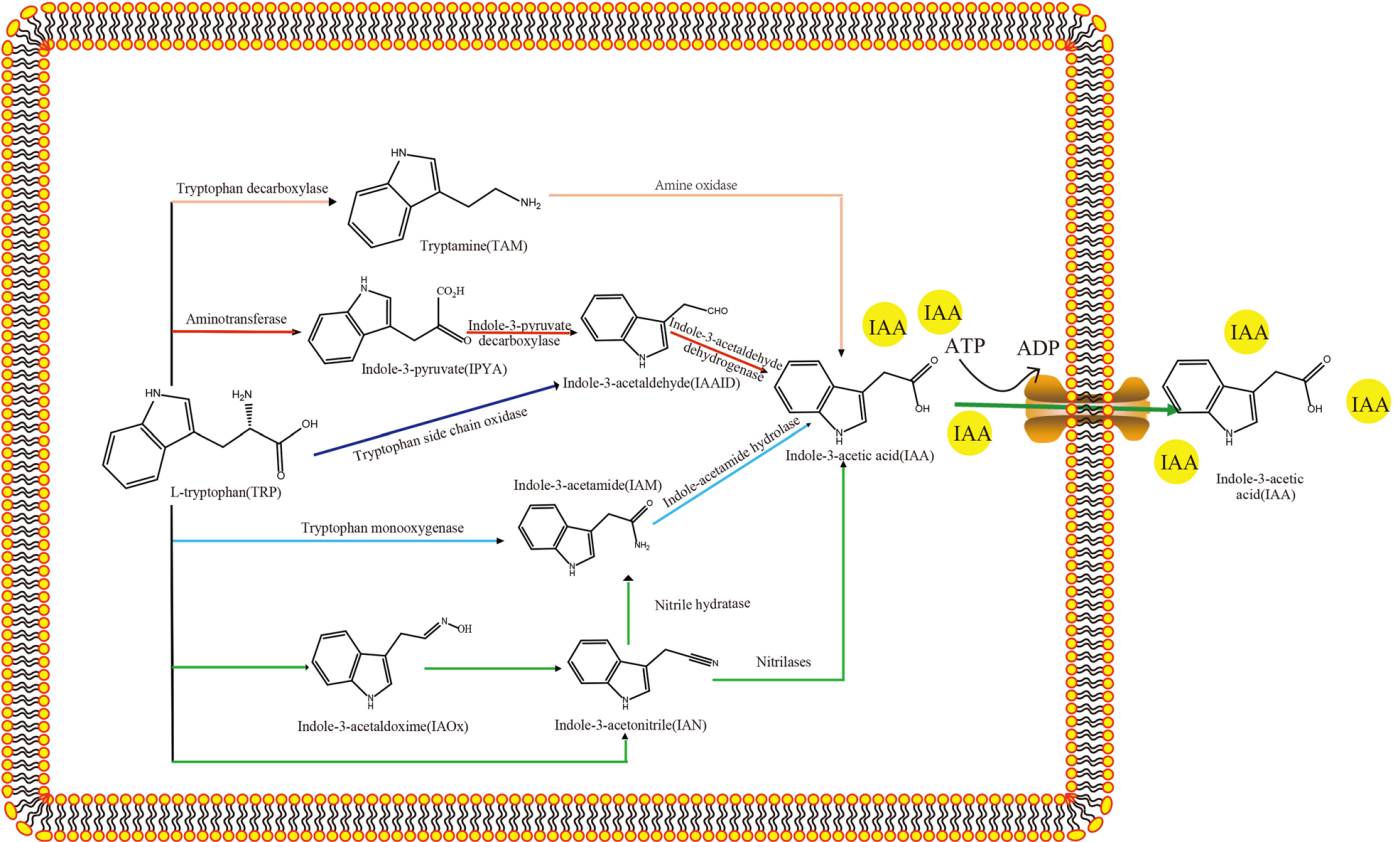
Five synthetic pathways of IAA depend on L-tryptophan in bacteria

IAA is the most important type of auxin, regulating plant growth and development [29, 30]. The IAA production in industrial application is now mainly synthesized by chemical method, but generally, numerous bacteria are capable of bioproducing IAA in a range of 10–250 μg/mL with or without 0.2–2 μg/mL of L-tryptophan [31]. IAA produced by PGPR can act in the rhizosphere of plants to directly stimulate root growth [32]. The IAA synthetic pathways of microorganisms are now gradually being analyzed and have mostly focused on a single specific gene of the IAA synthetic pathways [30]. Through functional analysis of genomes, enzymatic activities, observation of metabolic characteristics, and isotope label dilution tests, the IAA synthetic pathways of microorganisms might be divided into L-tryptophan-dependent and L-tryptophan-independent pathways [33, 34]. In bacteria, five potential L-tryptophan-dependent IAA synthetic pathways have been proposed (Fig. 1): the indole-3-pyruvic acid pathway (IPyA), indole-3-acetamide pathway (IAM), indole-3-acetonitrile pathway (IAN), tryptamine pathway (TAM), and tryptophan side-chain oxidase pathway (TSO) [35–37].

For the IPyA pathway of IAA synthesis, L-tryptophan is converted to indole-3-pyruvate by aminotransferase. Subsequently, indole-3-pyruvate is converted to indole-3-acetaldehyde (IAAlD) by indole-3-pyruvate decarboxylase (IPDC). Finally, indole-3-acetaldehyde dehydrogenase converts IAAlD to IAA [30]. Aminotransferases have been widely discovered in some Gram-negative bacteria, such as *Escherichia coli* and *Enterobacter cloacae* [38], and have also been reported in Gram-positive bacteria, such as *Bacillus amyloliquefaciens* SQR9 and *Arthrobacter pascens* ZZ21 [32, 39]*.* The encoding genes of the key enzyme IPDC have been widely identified and characterized in *Azospirillum brasilense*, *Agrobacterium tumefaciens*, *E. cloacae*, *Pseudomonas putida*, *Zymomonas mobilis*, *B. amyloliquefaciens*, *P. polymyxa*, etc*.* [29, 32, 38, 40]. Indole-3-acetaldehyde dehydrogenase genes have also been found in some bacteria, such as *E. coli*, *Pseudomonas syringae*, *A. brasilense*, *A. pascens*, and *B. amyloliquefaciens* [30, 34, 36, 37, 39]. The IAM pathway of IAA synthesis is the best-characterized pathway in bacteria which mainly exists in *Pseudomonas savastanoi* and *A. tumefaciens* [41–43]. The main genes driving the IAM pathway are *iaam* and *iaah*, which encode tryptophan monooxygenase and indole-3-acetamide hydrolase, respectively [44, 45]. Tryptophan monooxygenase catalyzes the conversion of L-tryptophan to indole-3-acetamide (IAM), and indole-3-acetamide hydrolase further catalyzes the hydrolysis of IAM to IAA [46]. The *iaam* and *iaah* genes were already found in *P. savastanoi* [47], *A. tumefaciens* [43], *Burkholderia pyrrocinia* [48], *Pseudomonas fluorescens* [39, 48], and some other strains. In regard to the IAN pathway of IAA synthesis, the biosynthesis genes have not been well characterized, and they might have two different pathways. For the first pathway of IAN, some studies have discovered that L-tryptophan can be directly converted to indole-3-acetonitrile (IAN) [49], and IAN can be further converted into IAA by the two-step enzymatic hydrolysis of nitrile hydratase and indole-3-acetamide hydrolase [50]. The reaction process involves nitrile hydratase catalyzing the conversion of IAN to IAM, and then indole-3-acetamide hydrolase hydrolyzing IAM into IAA [51]. For the second pathway of the IAN pathway, some studies have speculated that L-tryptophan can be converted to indole-3-acetaldoxime (IAOx), then IAOx is converted to IAN, and IAN is finally hydrolyzed into IAA by nitrilase [51]. To date, the enzymes responsible for the conversion of L-tryptophan to IAOx and the conversion of IAOx to IAN have been detected in bacteria [52]. Nitrilase was found in *E. coli* [53], *Rhodococcus ruber* [53], *P. fluorescens* [54], *B. amyloliquefaciens* [32], and other bacteria. In bacteria, the IAM and IAN pathways share the same indole-3-acetamide hydrolase to convert IAM into IAA. Nitrilase has not yet been detected in some strains of *Agrobacterium* and *Rhizobium* spp. [49], but these strains somehow have nitrilase activity. The TAM pathway of IAA synthesis has been suggested in *B. cereus* and *Azospirillum* by the identification of tryptophan decarboxylase activity [32, 55]. In this way, L-tryptophan is converted to tryptamine (TAM) by tryptophan decarboxylase and then converted to IAA by amine oxidase. The TSO pathway of IAA synthesis is a unique pathway that may only exist in *P. fluorescens* [56]. L-Tryptophan is converted to IAAlD under the catalysis of tryptophan side-chain oxidase [57] and then converted to IAA under the catalysis of indole-3-acetaldehyde dehydrogenase [51].

The IAA production and related genes of some Gram-negative bacteria have been widely studied; in contrast, the details of biosynthetic pathways utilized by Gram-positive bacteria remain less clear, and further research and exploration are still needed [37, 39]. As a Gram-positive PGPR, *P. polymyxa* can synthesize IAA to promote cell growth, division, and differentiation, and regulate the physiological functions of plants [37, 40, 58]. Some genes involved in the IAA synthetic pathways of *P. polymyxa* were analyzed as mentioned above, which were mainly focused on the IPDC encoding gene of the IPyA pathway. However, the other genes of the IAA synthetic pathways need further verification. In addition, auxin efflux carrier (AEC) proteins for IAA efflux were found and are widely present in the genome of *P. polymyxa* [28]. It has also been verified that a mutation in the *gpr1* gene could reduce the ability of *P. polymyxa* to synthesize IAA; however, mutations in the *relA/spoT* homologous gene and downstream of the *spo0F* gene could somehow increase the yield of IAA [59]. However, to date, the entire metabolic pathways and regulatory mechanisms of IAA synthesis in *P. polymyxa* have not been clearly identified, and the improvement of IAA production in *P. polymyxa* is of great value.

The genetic modification of *P. polymyxa* is beneficial for the characterization of its IAA synthetic pathways and the improvement of its application. The predictable control of gene expression is a main approach for genetic manipulation in *P. polymyxa* [60]. Promoters initiate the transcription process and play important roles in controlling gene expression [61, 62], which is an important strategy for metabolic engineering and synthetic biology research [63]. Previously, four heterogeneous promoters were tested for genetic expression in *P. polymyxa* [64]. Heinze et al. [65] evaluated 11 promoter sequences, which included well-characterized promoters from *Bacillus subtilis* and *Bacillus megaterium*, for the secretory production of a cellulase in *P. polymyxa* DSM292. Through high-throughput random screening, the native and continuously expressed promoter *P*_*LH-77*_ was also identified and characterized by our group [60]. The promoters described above are useful for heterologous expression in the host *P. polymyxa*. However, a series of powerful promoters with different activities have not yet been established in *P. polymyxa* for IAA production research. For predictable expression control of IAA synthetic genes in *P. polymyxa,* there is still no quantitative and strong gene expression system conducted by novel promoters with different expression intensities.

*P. polymyxa* SC2 was formerly isolated from rhizosphere soil of pepper and tested as an effective PGPR for increasing plant growth and having broad-spectrum antimicrobial activity by our group [66, 67], which can be used to produce microbial fertilizers. *P. polymyxa* SC2-M1 is a spontaneous mutant of strain SC2 with a high transformation capacity [68] and is an ideal material for molecular biology research on *P. polymyxa*. In this study, we identified that strain SC2-M1 retained the ability to synthesize IAA. A native IAA synthetic pathway of strain SC2-M1 was discovered, and the IAA yield was then enhanced through different metabolic pathways benefiting from a novel strong promoter, *P*_*04420*_.

## Results

### Identification of the IAA synthetic ability of *P. polymyxa* SC2-M1

**Fig. 2:**
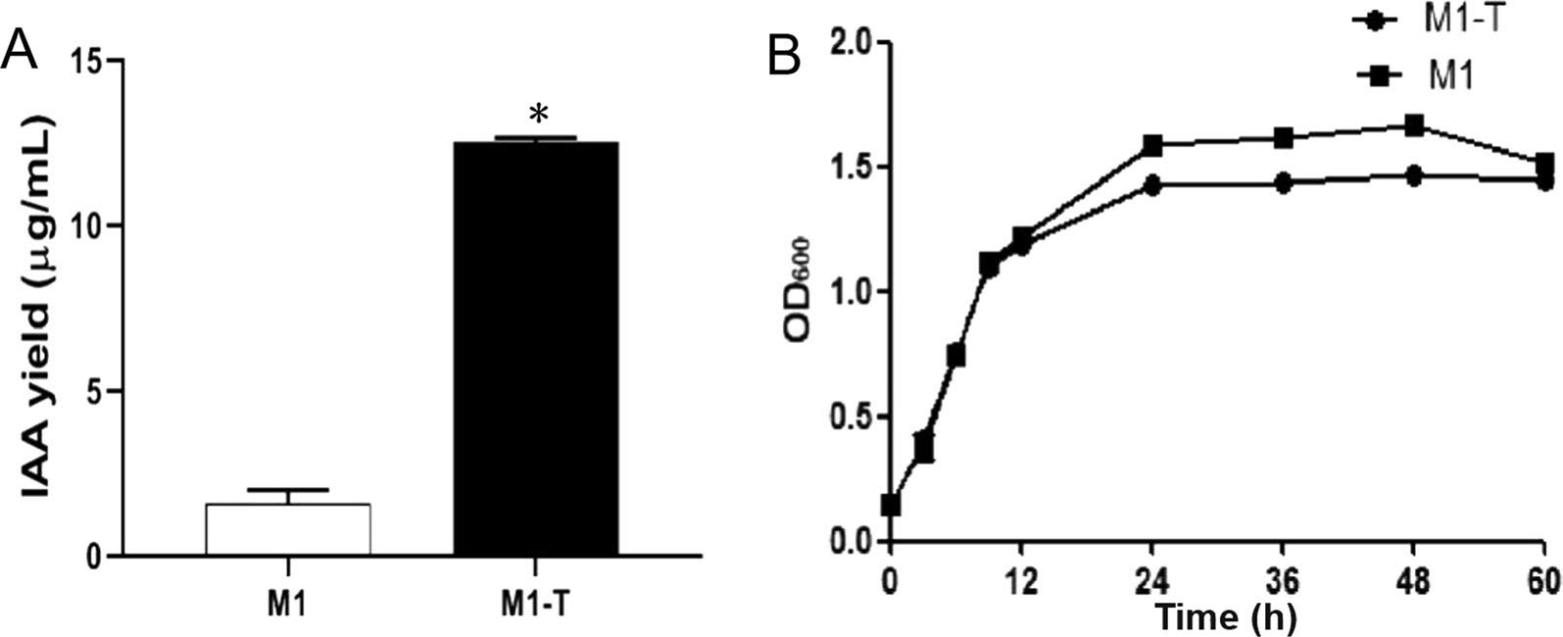
The IAA yield (**A**) and the growth curve (**B**) of *P. polymyxa* SC2-M1. Strain SC2-M1 was activated and inoculated by 5% in Landy medium with or without 3 mM L-tryptophan for 72 h at 25 °C. The concentration of IAA was measured by colorimetric method. The test group (named as M1-T) was cultured with 3 mM L-tryptophan compared with the control group (named as M1) without L-tryptophan. Compared with the control, data that significantly increased (*p* < 0.05) were marked with *

In the presence of inorganic acid, IAA can interact with FeCl_3_ to have color reaction and form a red chelate, which has a maximum absorption peak at 530 nm. Strain SC2-M1 was cultured in Landy medium both with and without L-tryptophan. The strains cultured without L-tryptophan had no color reaction, but the strains cultured with L-tryptophan had obvious color changes, indicating that strain SC2-M1 can produce IAA with L-tryptophan. The standard curve of IAA was measured as *Y* (IAA, μg/mL) = 30.439 × X (OD_530_) + 0.3165 (*R*^2^ = 0.9992). Therefore, when 3 mM L-tryptophan was added to the medium, the IAA yield of strain SC2-M1 was 12.5 μg/mL (Fig. 2A). Therefore, there is at least one IAA biosynthesis pathway that depends on L-tryptophan in the genome of strain SC2-M1.

### Metabolome analysis of strain SC2-M1 for discovering IAA biosynthesis pathways

#### Selection of sampling time

The growth ability of strain SC2-M1 in Landy medium with or without L-tryptophan and the changes in IAA yield were tested. Strain SC2-M1 could accumulate IAA after 9 h of incubation, and at this time, strain SC2-M1 was in the logarithmic phase, which was suitable for sampling (Fig. 2B). Combining the growth status and IAA production of strain SC2-M1, the strain cells incubated for 9 h were selected and prepared for metabolome analysis.

#### Screening of differential metabolites

**Fig. 3:**
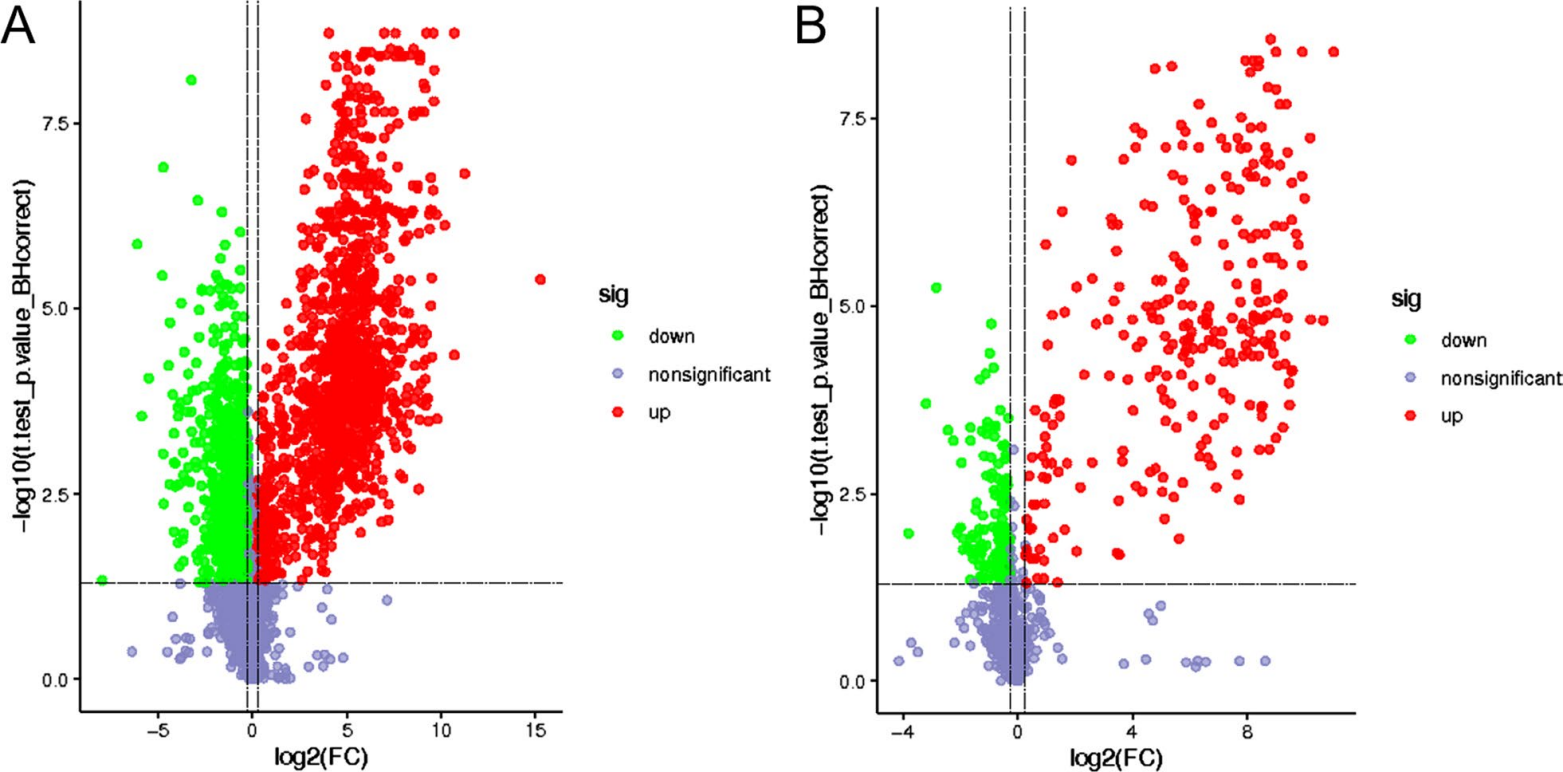
Volcano maps of differential metabolites in *P. polymyxa* SC2-M1. Differential metabolites in positive (**A**) and negative (**B**) ion mode. Green points represent the downregulated metabolites. Red points represent the upregulated metabolites. The non-different metabolites are marked as purple-grey points

Metabonomic analysis was performed on the test group containing 3 mM L-tryptophan (M1T) compared with the control group without 3 mM L-tryptophan (M1). The criteria of fold-change ≥ 1.2 or ≤ 0.83 and p value < 0.05 were used to screen the differential metabolites. After data preprocessing, the total number of compounds and the number of differential metabolites were statistically analyzed according to the positive and negative ion modes, as shown in the volcano diagram in Fig. 3. In the positive ion mode (Fig. 3A), a total of 2224 differential metabolites were identified, of which 1286 metabolites were upregulated, 938 metabolites were downregulated, and 998 metabolites had known specific functions. In the negative ion mode (Fig. 3B), a total of 485 differential compounds were identified, of which 280 metabolites were upregulated, 205 metabolites were downregulated, and 227 metabolites had known specific functions. The differential metabolites that were identified were mainly enriched in the positive ion mode, and there were far more upregulated metabolites than downregulated metabolites.

#### Cluster and enrichment analysis of differential metabolites

**Fig. 4:**
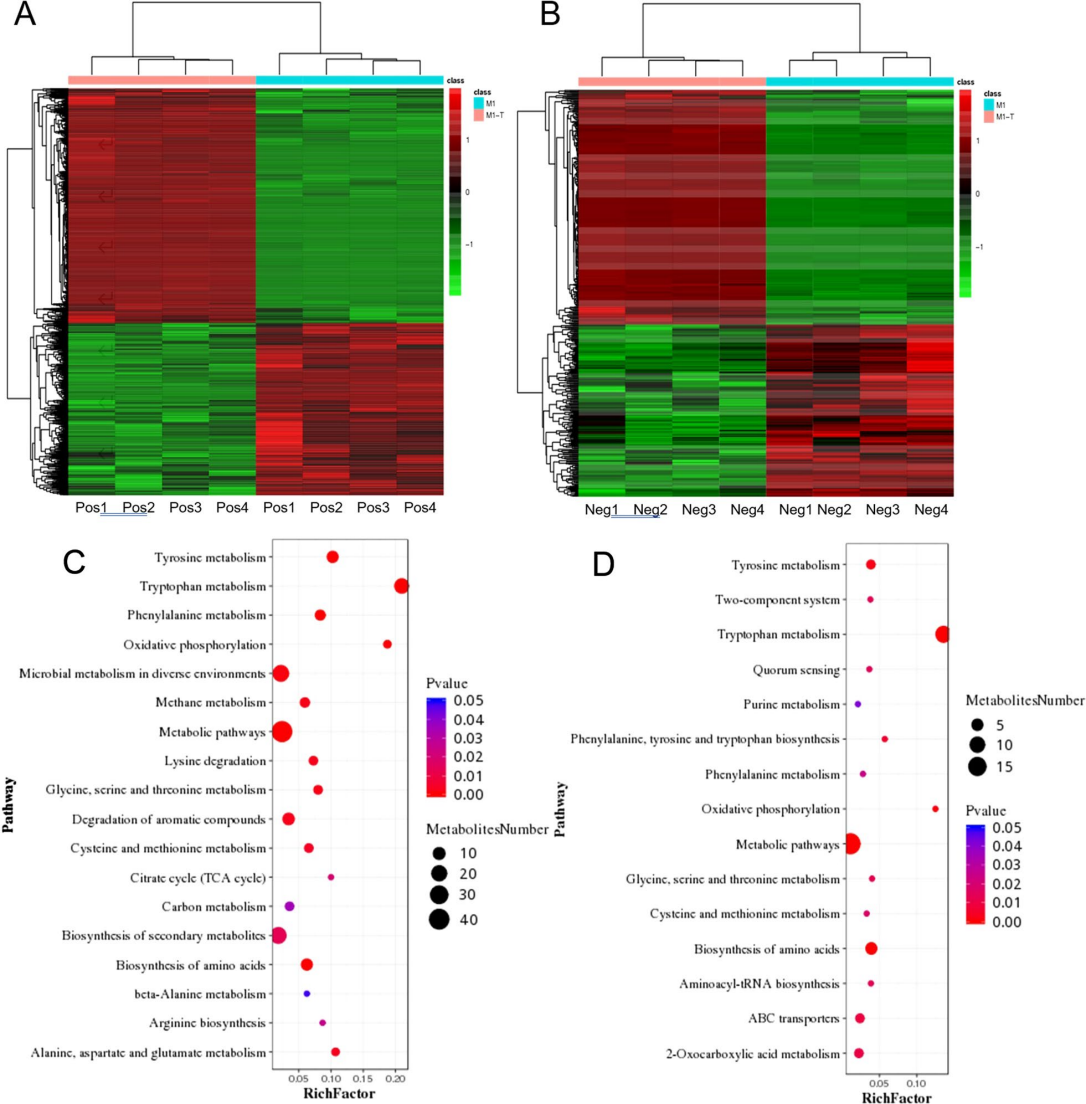
Classification and analysis of differential metabolites in *P. polymyxa* SC2-M1. Cluster analysis of differential metabolites in the positive (**A**) and negative (**B**) ion mode. Each row represents a differential metabolite and each column represents a sample. The color represents the expression level of differential metabolites, and the green to red corresponds to the expression level from low to high. Bubble chart of metabolic pathway enrichment analysis in the positive (**C**) and negative (**D**) ion mode. Red represents the significant enrichment and the size of the dot represents the number of different metabolites annotated in the pathway

To understand the classification and functional characteristics of the different metabolites for the test Group M1T vs. the control Group M1, GO annotation and KEGG functional analysis were performed on the identified metabolites. The results demonstrated that most of the differential metabolites were primarily concentrated in the biochemical metabolic pathways and signal transduction pathways. Among them, 166 metabolites were enriched in positive ion mode, and 62 metabolites were enriched in negative ion mode. The cluster analysis of the metabolites in these samples is shown in Fig. 4. In the positive ion mode (Fig. 4A) and negative ion mode (Fig. 4B), the overall Euclidean distances among the 4 parallel samples of the control group or the 4 parallel samples of the test group are very small, which indicates that the parallelism among the 4 parallel samples in the two groups is good. The results of our analysis are based on the synthesis of the 4 parallel samples in the two groups. The same metabolite can show different enrichment effects under different ion modes. Combined with the analysis of positive and negative ion models, there is a certain overlap for a certain metabolite in the control group or test group, indicating that the metabolic pathways involved in this metabolite are significantly enriched.

Under the condition of *p* value < 0.05, the KEGG database was employed to combine the metabolic pathway enrichment analysis in a bubble chart to screen the metabolic pathways with significant enrichment of differential metabolites (Fig. 4C and D, and Additional file 1: Table S2). In the positive ion mode (Fig. 4C), differential metabolites were enriched in 18 metabolic pathways, of which 17 counts were enriched in tryptophan metabolic pathways, 22 counts were enriched in microbial metabolism in diverse environments, 22 counts were enriched in biosynthesis of secondary metabolites, 9 counts were enriched in degradation of aromatic compounds, and 8 counts were enriched in tyrosine metabolism and other metabolic pathways. In the negative ion mode (Fig. 4D), differential metabolites were enriched in 15 metabolic pathways, of which 11 counts were enriched in the tryptophan metabolic pathways, 5 counts were enriched in the biosynthesis of amino acids, and a few counts were enriched in the biosynthesis of aminoacyl-tRNA biosynthesis, tyrosine metabolism, the ABC transporter, etc. Comprehensive analysis of the enriched metabolites in both positive and negative ion modes showed that differential metabolites in tryptophan metabolic pathways were significantly enriched in more than 10 counts in the two modes. The differential metabolites in tryptophan metabolic pathways are closely related to IAA biosynthesis.

#### Analysis of metabolites involved in L-tryptophan metabolism pathways

The different metabolites related to the IAA biosynthetic pathways were screened and the related genes were revealed in strain SC2-M1. Through KEGG analysis, it was found that the intermediate metabolites (Group M1T vs. Group M1) in the tryptophan metabolic pathways related to the IAA biosynthesis process were increased, such as indole, tryptamine, indole-3-lactic acid, indole pyruvate, indole-3-ethanol, indole-3-acetamide, and IAA. The results proved that strain SC2-M1 has IAA biosynthetic pathways depending on L-tryptophan, which can be further explored.

#### Screening of genes likely to be involved in the IAA biosynthetic pathways of *P. polymyxa* SC2-M1

Screening of genes likely to be involved in the IAA biosynthetic pathways of *P. polymyxa* SC2-M1 was done by combining the metabolome results and genome annotation information. Based on the proposed IAA biosynthetic pathways that depend on L-tryptophan in bacteria [46, 69], the entire *P. polymyxa* SC2-M1 genome was mined for genes involved in each step of different IAA biosynthetic pathways (Table 1). The candidate genes were screened according to their deduced amino acid sequences with enzymes that were already known in IAA metabolic pathways. In regard to the discovery of the IPyA pathway, the genes *patA*, *alaT1*, and *ykrV1* may encode aminotransferase; the genes *ilvB3*, *poxB*, *pdhA*, and *pdhB* may be involved in the indole-3-pyruvate decarboxylase reaction; and the final step is the conversion of indole-3-acetaldehyde to IAA catalyzed by indole-3-acetaldehyde dehydrogenase, which may be encoded by the *fusE* and *adhE* genes. In regard to the discovery of the TAM pathway, the genes coding for tryptophan decarboxylase were not detected in strain SC2-M1. However, the gene *sdr2* encoding amine oxidase in the second step of the TAM pathway was discovered. In regard to the most common IAM pathway in bacteria, the *iaam* gene encoding tryptophan monooxygenase was not detected in the genome of strain SC2-M1, but the genes *gatA1*, *gat* and *yhaA1*, which hold high homology with the indole-3-acetamide hydrolase coding gene *iaah*, exist in the genome of strain SC2-M1. In regard to the possible IAN pathway, the *PPSC2_05390* gene encoding nitrile hydratase and the *nit2* gene encoding nitrilase were found in the genome of strain SC2-M1, but the related activities need to be tested. The genes encoding indole-3-acetamide hydrolase in the IAN pathway are the same as those in the IAM pathway.

**Table 1:** The predicted native IAA synthetic pathways of *P. polymyxa* SC2-M1

IAA synthetic pathway	Enzyme	Related gene
IPyA	Aminotransferase	*patA* (*PPSC2_17445)*, *alaT1* (*PPSC2_07190*), *ykrV1 (PPSC2_14305*)
	Indole-3-pyruvate decarboxylase	*ilvB3* (*PPSC2_07070*), *poxB* (*PPSC2_10740*), *pdhA* (*PPSC2_13545*), *pdhB* (*PPSC2_13540*)
	Indole-3-acetaldehyde dehydrogenase	*fusE* (*PPSC2_00395*), *adhE* (*PPSC2_15245*)
TAM	Tryptophan decarboxylase	Not detected
	Amine oxidase	*sdr2* (*PPSC2_12320*)
IAM	Tryptophan monooxygenase	Not detected
	Indole-3-acetamide hydrolase	*gatA1* (*PPSC2_07840*), *gat* (*PPSC2_12215*), *yhaA1* (*PPSC2_13350*)
IAN	Nitrile hydratase Indole-3-acetamide hydrolase	*PPSC2_05390* *gatA1* (*PPSC2_07840*), *gat* (*PPSC2_12215*), *yhaA1* (*PPSC2_13350*)
	Nitrilase	*nit2* (*PPSC2_14300*)

**Fig. 5:**
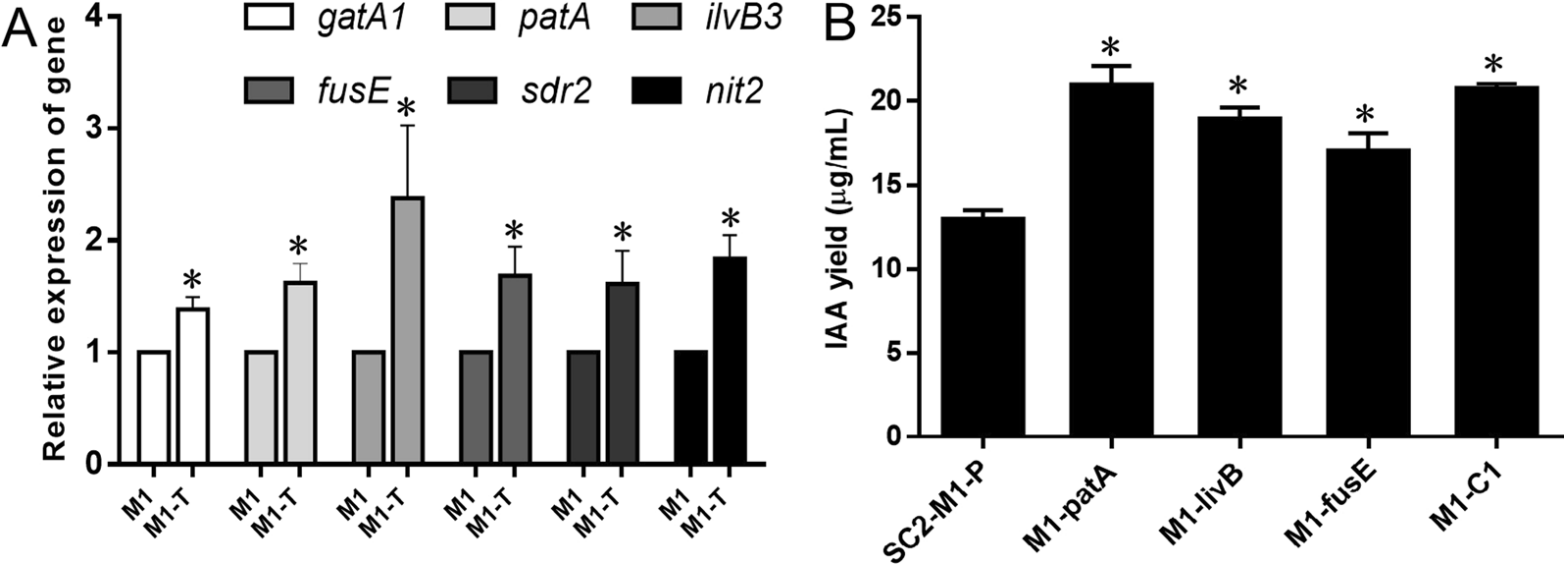
Relative expression levels of genes induced by L-tryptophan and the IAA yield of modified strain SC2-M1. Relative expression levels of genes *gatA1*, *patA*, *ilvB3*, *fusE*, *sdr2*, and *nit2* in the logarithmic phase of strain SC2-M1 in the test group (named as M1-T) that was cultured with 3 mM L-tryptophan compared with the control group (named as M1) without L-tryptophan (**A**). The IAA yield of strain SC2-M1 overexpressing genes *patA*, *ilvB3*, and *fusE* (**B**). The IAA yields were tested by the colorimetric method and adding 3 mM L-tryptophan in Landy medium for 72 h at 25 °C. SC2-M1-P is the control strain containing empty plasmid pHY300PLK, Strains M1-patA, M1-ilvB, and M1-fusE represent genes *patA*, *ilvB3*, and *fusE* overexpressing strains, respectively. Strain M1-C1 represents *patA-ilvB3-fusE* co-expressing strain. Compared with the control, data that significantly increased (*p* < 0.05) were marked with *

Since all of the candidate genes (Table 1) were potentially involved in L-tryptophan-dependent IAA biosynthesis of strain SC2-M1, the active genes were further identified via transcriptional responses by the addition of L-tryptophan. When 3 mM L-tryptophan was added to the medium, six of the candidate genes were found to be significantly induced by L-tryptophan (Fig. 5A): the mRNA relative expression of the genes *gatA1*, *patA*, *ilvB3*, *fusE*, *sdr2*, and *nit2* was increased by 38%, 62%, 138%, 69%, 61%, and 84%, respectively. These six genes were proposed to be involved in the tryptophan-dependent IPyA, TAM, IAN, and an uncharacterized IAA biosynthesis pathway. This finding indicates that multiple IAA biosynthesis pathways exist in *P. polymyxa* SC2-M1, and a set of the entire genes in the IPyA pathway were all identified.

### Screening and identification of endogenous and high-efficiency promoters for expressing and verifying genes involved in the IAA synthetic pathways

#### Rational identification of native promoters resulting from transcriptome data in strain SC2-M1

The availability of endogenous promoters with different transcription levels in *P. polymyxa* to control gene expression is still necessary. Moreover, the basic structures of the endogenous promoters of *P. polymyxa* have not been revealed. Due to the former transcriptome data of strain SC2-M1 [68] on LB media, we selected 77 potential promoters of expressed genes with high average values of RPKM values (reads per kilobase of transcript per million reads mapped) to analyze the basic structures of endogenous promoters of strain SC2-M1. The − 35 and − 10 regions of these promoters were predicted by Softberry-BPROM. Then, the − 35, − 10, and RBS regions, and their flanking sequences of these promoters were analyzed using the WEBLOGO website (Additional file 1: Fig. S1). The spacer bases between − 35 and − 10 regions were filled with “–” when less than 22 nt. For the promoter sequences of the 77 highly transcribed genes of strain SC2-M1 under normal growth conditions on LB medium, the conserved bases of the − 35 and − 10 regions were “TTG(A/C)NN” and “TA(T/A)AAT”, respectively. The resulting − 10 and − 35 regions were close to the consensus recognition sequences of bacteria reported [70, 71]. The conserved bases of the RBS region were purine-rich bases “G/A” from the 9th base to the 14th base before the start codon. Analyzing the conservative bases of the promoter sequences is beneficial for understanding the favorable regions of native promoters in strain SC2-M1 and this might be easier or consumes less energy to express genes.

**Fig. 6:**
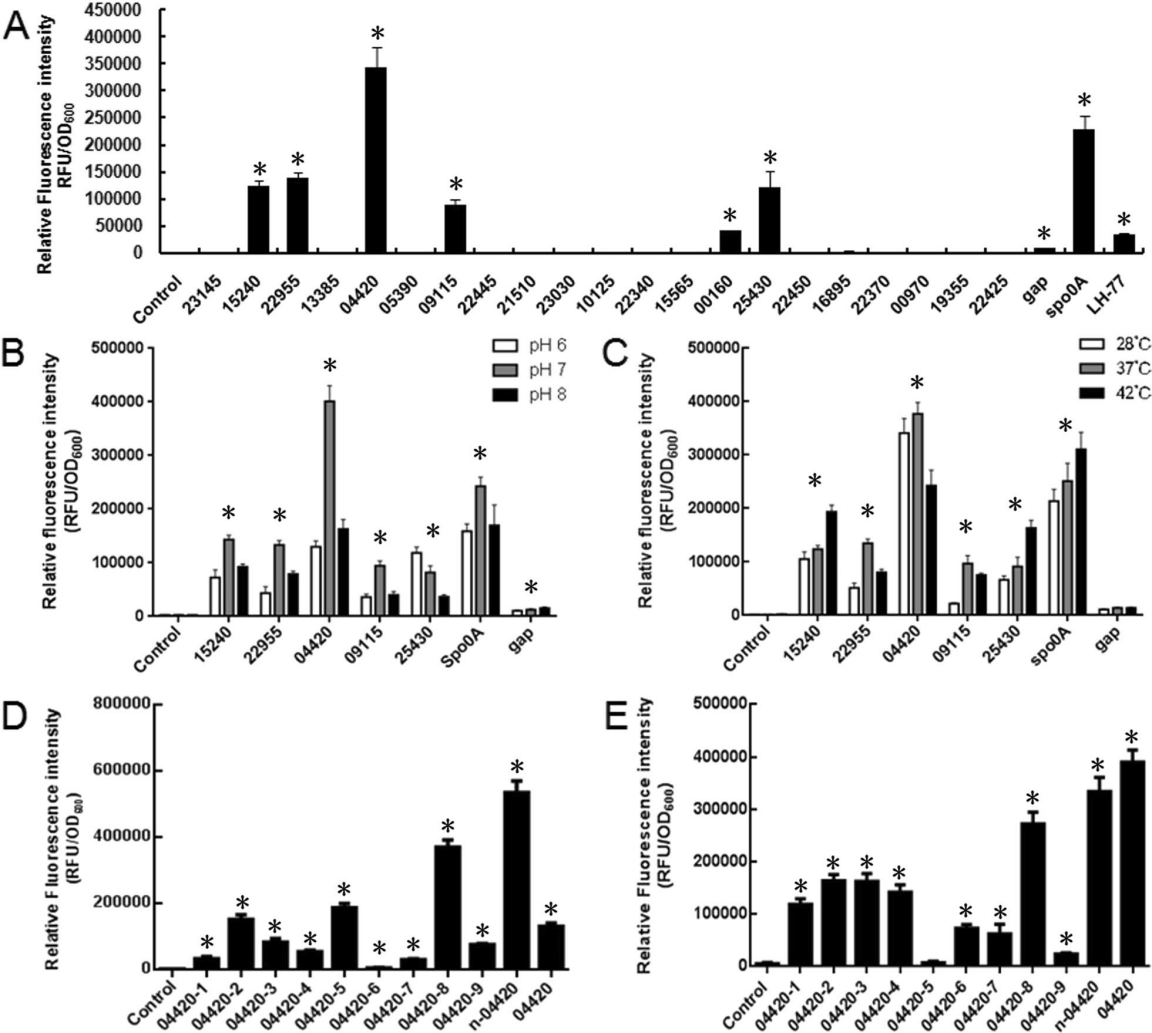
Analysis of promoter strength in *P. polymyxa* SC2-M1 or *E. coli* DH5α. Relative fluorescence intensities of different promoters expressing GFP in *P. polymyxa* SC2-M1 (**A**)*.* Relative fluorescence intensities of endogenous strong promoters expressing GFP in *P. polymyxa* SC2-M1 under different pH (**B**) and temperature (**C**). Relative fluorescence intensities of engineered *P*_*04420*_ expressing GFP in *E. coli* DH5α (**D**) and strain SC2-M1 (**E**). Single colonies of fresh strains were preincubated on LB liquid medium, and then transferred to fresh media to a final concentration of 10% for 24 h incubation. Control contains no promoter for GFP expression. Compared with the control, data that significantly increased (*p* < 0.05) were marked with *

The promoter data of 25 genes arranged in descending order according to the average RPKM value are shown in Additional file 1: Table S3. We selected the promoters corresponding to these genes to study their expression activity. Among these 25 genes, 7 genes were distributed to three groups with polycistronic relationships. Thus, based on the transcriptome data of strain SC2, a total of 21 promoters were selected. In addition, the promoter *P*_*spo0A*_ of the sporulation transcription factor Spo0A was also rationally selected, so we rationally selected out 22 promoters. The selected 22 promoters were then verified in *P. polymyxa* SC2-M1. A constitutive promoter *P*_*gap*_ in *Paenibacillus* [64] and a promoter *P*_*LH-77*_ that was formerly reported by our group [60] were selected as positive controls in this study. Compared with the negative control, there were seven strong promoters in *P. polymyxa* SC2-M1, and the order of promoter activities from high to low was *P*_*04420*_ > *P*_*spo0A*_ > *P*_*22955*_ > *P*_*25430*_ > *P*_*15240*_ > *P*_*09115*_ > *P*_*00160*_ (Fig. 6A). The strength of the six promoters was higher than that of *P*_*LH-77*_ [60], and the activities of promoters *P*_*00160*_ and *P*_*LH-77*_ were similar. The microscopic fluorescence observation of GFP expressed by the six promoters *P*_*15240*_, *P*_*22955*_, *P*_*04420*_, *P*_*09115*_, *P*_*25430*_, and *P*_*spo0A*_ in strain SC2-M1 is also presented in Additional file 1: Fig. S2, and these six endogenous strong promoters were selected for further research.

In addition, as *B. subtilis* 168 is a Gram-positive model organism, the selected promoters with high strength were also expressed in *B. subtilis* 168 to verify their general applicability in Gram-positive bacteria. Compared with the control, promoters *P*_*04420*_, *P*_*spo0A*_, *P*_*22955*_, *P*_*15240*_, and *P*_*09115*_ also worked well in *B. subtilis* 168 (Additional file 1: Fig. S3). We also tested the relative fluorescence intensities of different promoters expressing GFP in Gram-negative *E. coli* DH5α, and the activities of these promoters showed a different trend in the Gram-negative background (Additional file 1: Fig. S3). It is worth noting that *P*_*04420*_ was the strongest in *E. coli*, similar to strain SC2-M1.

#### Effects of different pH values and temperatures on the expression of endogenous strong promoters in strain SC2-M1

We set different pH and temperature conditions to identify the expression stability of the six promoters *P*_*04420*_, *P*_*spo0A*_, *P*_*22955*_, *P*_*25430*_, *P*_*15240*_, and *P*_*09115*_. The constitutive promoter *P*_*gap*_ was used as a positive control. The pH value of the LB media was adjusted to 6, 7, and 8 using HCl or NaOH solutions. As shown in Fig. 6B, the activities of *P*_*gap*_ did not change with pH value. However, when strain SC2-M1 was in an acidic or alkaline environment, the expression activity of these endogenous strong promoters also changed accordingly. The expression activity of *P*_*25430*_ decreased with increasing pH and was suitable for gene expression under acidic conditions. The expression activities of the remaining promoters were more suitable for neutral conditions (pH = 7). The average expression activity of *P*_*04420*_ was the highest across the three pH conditions. To determine the expression stability of the six endogenous strong promoters in different temperature environments, strain SC2-M1 was grown at 28 °C, 37 °C, and 42 °C. As shown in Fig. 6C, *P*_*gap*_ did not change with temperature. The expression activities of the promoters at 28 °C were all lower than those at 37 °C. Moreover, the expression activities of *P*_*15240*_, *P*_*25430*_, and *P*_*spo0A*_ at 42 °C were better than those at 37 °C. In contrast, the expression activities of *P*_*22955*_, *P*_*04420*_, and *P*_*09115*_ were better at 37 °C. The average expression activity of *P*_*04420*_ was the highest across the three temperatures.

#### Sequence modification and activity characterization of promoter *P*_*04420*_

**Fig. 7:**
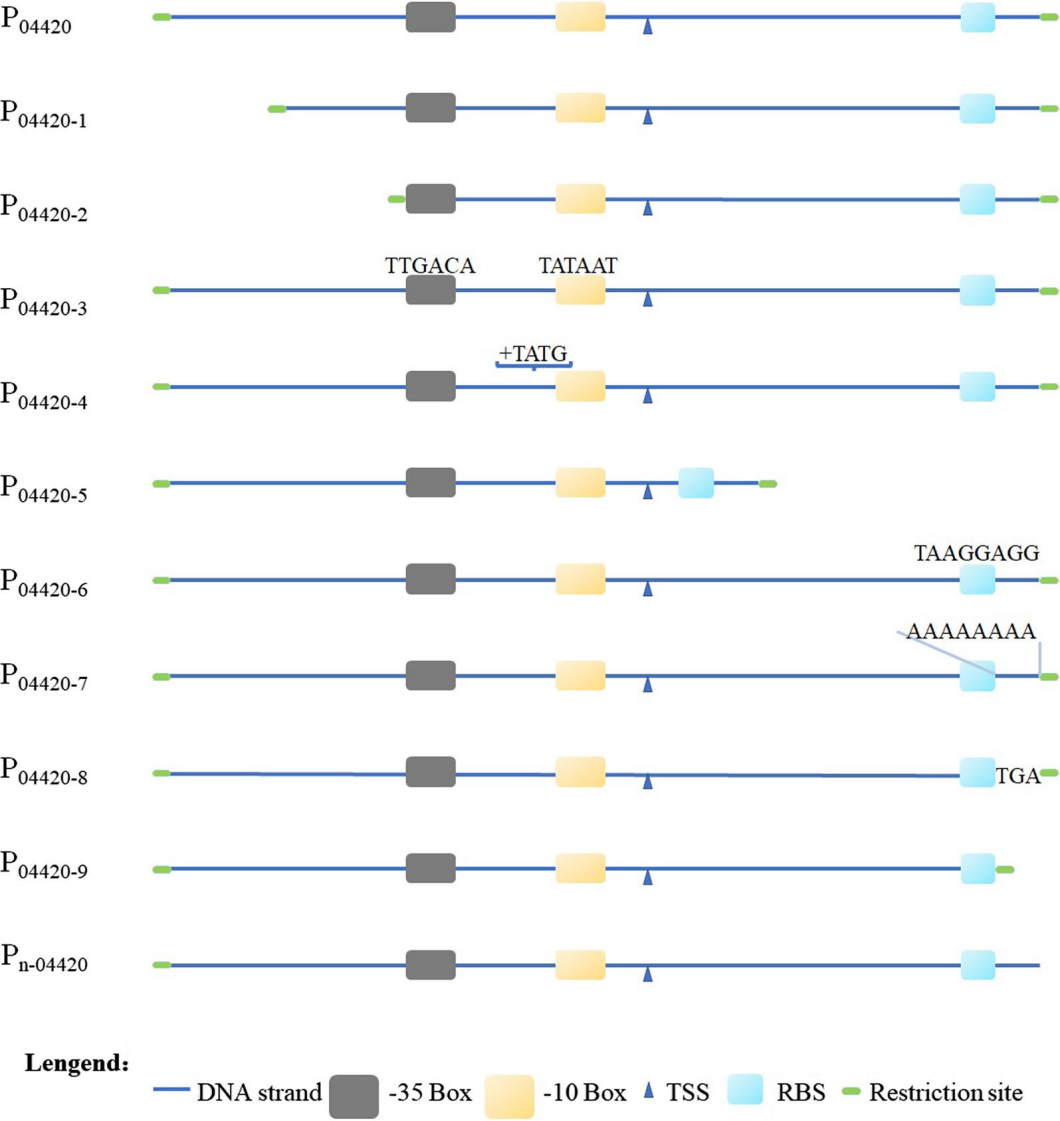
Sequence diagram of ten derivatives of promoter *P*_*04420*_. The changed sequences were indicated in the corresponding sites

Through the above experiments, several endogenous strong promoters in strain SC2-M1 were determined and the expression activity of *P*_*04420*_ was found to be relatively ideal, so *P*_*04420*_ was selected to artificially modify the activity of the promoter. First, the upstream element sequence, core promoter region, downstream element sequence, − 35, − 10, and RBS regions of *P*_*04420*_ were predicted. According to the characteristics of sequences in prokaryotic promoters, we used the idea of synthetic biology to design and modify the different sequence regions in *P*_*04420*_ by deleting, adding, or replacing bases in the promoter sequence. A total of 10 modified promoters of *P*_*04420*_ were obtained: *P*_*04420-1*_, *P*_*04420-2*_, *P*_*04420-3*_, *P*_*04420-4*_, *P*_*04420-5*_, *P*_*04420-6*_, *P*_*04420-7*_, *P*_*04420-8*_, *P*_*04420-9*_, and *P*_*n-04420*_. A schematic diagram of the design of the *P*_*04420*_ sequence is shown in Fig. 7. The arrangement of the original sequence of *P*_*04420*_ and the characteristics of the modified promoter sequence are shown in Additional file 1: Table S4. *P*_*04420-1*_ represents the sequence in which the upstream element sequence of *P*_*04420*_ is truncated by half; *P*_*04420-2*_ represents the sequence of *P*_*04420*_ with the upstream element removed completely; *P*_*04420-3*_ represents the sequence of the -35 and -10 regions of *P*_*04420*_ that were mutated into the sequences of TTGACA and TATAAT, respectively; *P*_*04420-4*_ represents the sequence with four bases of TATG added between the − 35 and − 10 regions of *P*_*04420*_; *P*_*04420-5*_ represents the sequence of *P*_*04420*_ with the downstream sequence truncated; *P*_*04420-6*_ represents the RBS sequence of *P*_*04420*_ that was mutated to the sequence TAAGGAGG; the 8 bases between the RBS sequence of *P*_*04420*_ and the *Bam*H I restriction site were replaced with the sequence AAAAAAAA to obtain *P*_*04420-7*_; the 8 bases between the RBS sequence of *P*_*04420*_ and the *Bam*H I site were replaced with the sequence TGA to obtain *P*_*04420-8*_; the sequence of *P*_*04420-9*_ represents the *P*_*04420*_ sequence with the 8 bases between the RBS sequence and the *Bam*H I sequence deleted; *P*_*n-04420*_ is the sequence of *P*_*04420*_ with the *Bam*H I restriction site removed between RBS and ORF.

The expression activities of each modified promoter of *P*_*04420*_ in *E. coli* (Fig. 6D) and strain SC2-M1 (Fig. 6E) were both determined at pH = 7 and 37 °C. The expression activities of these promoters in strain SC2-M1 were ranked as follows: *P*_*04420*_ > *P*_*n-04420*_ > *P*_*04420-8*_ > *P*_*04420-2*_ > *P*_*04420-3*_ > *P*_*04420-4*_ > *P*_*04420-1*_ > *P*_*04420-6*_ > *P*_*04420-7*_ > *P*_*04420-9*_ > *P*_*04420-5*_. The expression activities of the ten modified promoters all showed different degrees of downregulation compared with *P*_*04420*_. The modified promoter *P*_*04420-5*_ was almost inactive. According to the different expression activities of all modified promoters and *P*_*04420*_ in strain SC2-M1, the influence of these modified regions on the promoter activity could be observed. The changes in the upstream sequence of *P*_*04420*_ (*P*_*04420-1*_, *P*_*04420-2*_, *P*_*04420-3*_, and *P*_*04420-4*_) attenuated the promoter activity. The changes in the downstream sequence of *P*_*04420*_ (*P*_*04420-5*_, *P*_*04420-6*_, *P*_*04420-7*_, *P*_*04420-8*_, *P*_*04420-9*_, and *P*_*n-04420*_) also attenuated promoter activities, and the degree of influence was greater than that of upstream sequence. To further explore whether the expression activities of the modified promoters in strain SC2-M1 were consistent with those in *E. coli*, we transformed the 10 modified promoters into *E. coli* DH5α; however, their activities in *E. coli* were inconsistent with those in strain SC2-M1. In *E. coli*, the expression activities of these promoters in descending order were *P*_*n-04420*_ > *P*_*04420-8*_ > *P*_*04420-5*_ > *P*_*04420-2*_ > *P*_*04420*_ > *P*_*04420-3*_ > *P*_*04420-9*_ > *P*_*04420-4*_ > *P*_*04420-1*_ > *P*_*04420-7*_ > *P*_*04420-6*_. There were 4 modified promoters whose expression activity was upregulated. The most active promoter, *P*_*n-04420*_ was approximately 4 times higher than that of *P*_*04420*_; there were 6 downregulated promoters. The modified promoter *P*_*04420-6*_ had the lowest activity, which showed that RBS was the key region of promoter *P*_*04420*_ for expressing genes in *E. coli.*

#### The application of different derivatives of *P*_*04420*_ to express α-amylase

α-Amylase is an important industrial enzyme that is mainly cloned from microorganisms. Through the above experiments, we obtained several promoters with different activities. The corresponding relationship between promoter expression activity and enzyme activity was verified by further expressing α-amylase derived from strain SC2-M1. In this part, we selected 5 modified promoters of *P*_*04420*_ with different activities (*P*_*04420-4*_, *P*_*04420-6*_, *P*_*04420-8*_, *P*_*04420-9*_*,* and *P*_*04420*_) to drive the overexpression and expression of the α-amylase gene in strain SC2-M1 and *E. coli* DH5α, respectively. The α-amylase activity expressed by each promoter in different hosts was observed by transparent circle experiments. The recombinant bacteria fused with the empty plasmid pHY300PLK were used as the negative control (Additional file 1: Fig. S4). In strain SC2-M1, the α-amylase expression activities of the 5 modified promoters in descending order were *P*_*04420*_ > *P*_*04420-8*_ > *P*_*04420-4*_ > *P*_*04420-6*_ > *P*_*04420-9*_. When expressing extracellular α-amylase, the transparent circle radiuses of the *P*_*04420*_, *P*_*04420-8*_*,* and *P*_*04420-4*_ expressing strains were similar; *P*_*04420-6*_ had weaker activity; and *P*_*04420-9*_ had no activity, as its clear circle was smaller than that of the negative control. Although the activities of the promoters *P*_*04420*_ and *P*_*04420-8*_ were higher than those of *P*_*04420-4*_, the activities of the expressed extracellular α-amylase were similar. The promoter activity of *P*_*04420-4*_ was high enough to express extracellular α-amylase in strain SC2-M1. Similarly, the above five promoters could also express extracellular α-amylase in *E. coli* and promoter *P*_*04420-6*_ had the lowest α-amylase activity (Additional file 1: Fig. S4).

Taken with the above results, when a promoter reaches a certain strength, the enzyme activity expressed reaches a peak value, which might be due to the cell restriction of a limitation of endogenous substances in cells and the cellular homeostasis. It was also shown that the recombinant strain caused certain metabolic pressure for heterologous expression or overexpression of α-amylase, so it was necessary to select a promoter with suitable activity. The endogenous α-amylase gene was successfully expressed and exhibited biological activities in strain *P. polymyxa* SC2-M1 using promoter *P*_*04420*_. It was confirmed that promoter *P*_*04420*_ and its derivatives could be utilized to perform gene expression of IAA synthesis in *P. polymyxa*.

#### Verification of the endogenous IAA synthetic pathways of *P. polymyxa* SC2-M1 by gene overexpression using the promoter *P*_*04420*_

Based on metabolome analysis, fluorescence quantitative results (Fig. 5A), and the whole-genome protein sequences, the genes *patA*, *ilvB3*, and *fusE* might constitute an entire IPyA pathway of IAA synthesis in strain SC2-M1. The overexpression of *patA*, *ilvB3*, and *fusE* and the co-overexpression of *patA-ilvB3-fusE* by *P*_*04420*_ in *P. polymyxa* SC2-M1 increased IAA yield by 62%, 46%, 32%, and 60%, respectively (Fig. 5B)*.* The overexpression of the related genes *patA*, *ilvB3*, and *fusE* could strengthen the IAA synthesis pathway and further increase the IAA yield of *P. polymyxa* SC2-M1. The IAA yield of the co-expression strain M1-C1 increased to 20.8 μg/mL, only indicating an increase of 60%, which was almost the same as the overexpression of the first key gene, *patA*. Moreover, the presence of the extra plasmid pHY300PLK in strain SC2-M1 (Fig. 5B) did not significantly affect the IAA yield compared with strain SC2-M1 without the plasmid (Fig. 2A). To further enhance the IAA synthetic ability of strain SC2-M1, more powerful related genes of the IPyA pathway or other IAA synthetic pathways should be considered. Furthermore, the genes *patA*, *ilvB3*, and *fusE* were also heterogeneously co-overexpressed in *E. coli*, and we detected a small yield of IAA (~ 2.9 µg/mL).

#### Construction and modification of heterologous IAA pathways in *P. polymyxa* SC2-M1

The IAM and IPyA pathways are the main IAA synthesis pathways in bacteria [72]. From the above results, overexpression of the native IPyA pathway increased the IAA yield of strain SC2-M1 (Fig. 5B), but not by enough. Therefore, constructing heterologous IAM and IPyA pathways might further strengthen the IAA synthesis ability of strain SC2-M1.

#### Overexpression of a heterologous IAM pathway of IAA synthesis in strain SC2-M1

**Fig. 8:**
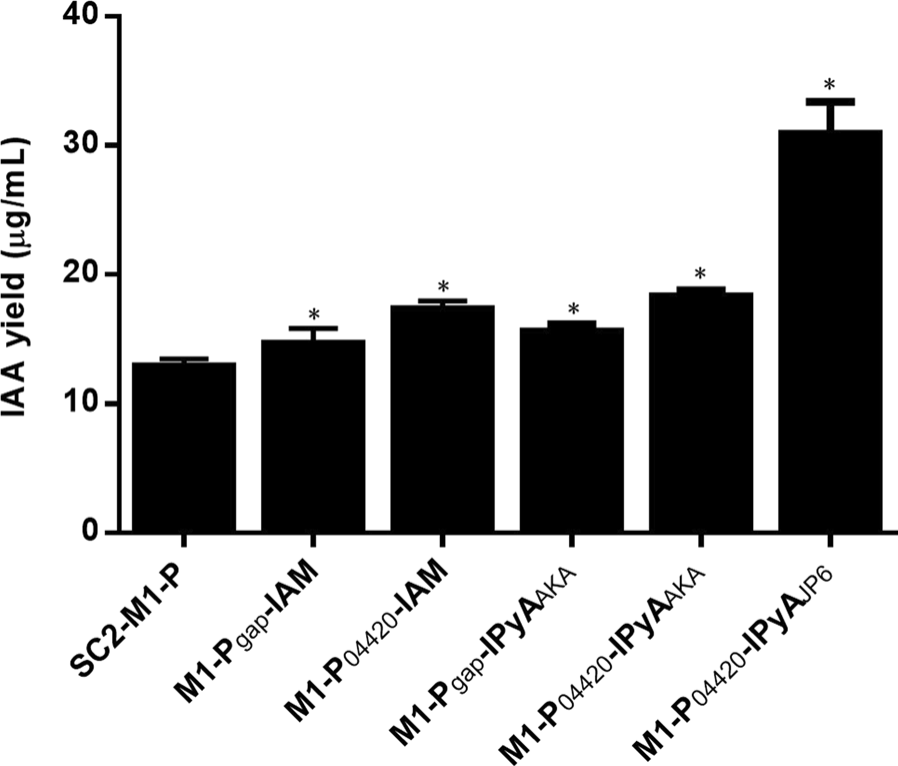
The IAA yield of strain SC2-M1 expressing heterologous IAA pathways. SC2-M1-P represents the control containing empty pHY300PLK. M1-P_gap_-IAM and M1-P_04420_-IAM represent transformants of strain SC2-M1 expressing the heterologous gene *iaam* by promoter *P*_*gap*_ and *P*_*04420*_, respectively. M1-P_gap_-IPyA_AKA_ and M1-P_04420_-IPyA_AKA_ represent transformants of strain SC2-M1 expressing heterologous genes *aro8, kdc,* and *aldH* by promoter *P*_*gap*_ and *P*_*04420*_, respectively. M1-P_04420_-IPyA_JP6_ represents the transformant of strain SC2-M1 expressing heterologous genes *ELJP6_14505*, *ipdC*, and *ELJP6_00725* by promoter *P*_*04420*_*.* IAA yields were tested by the colorimetric method and adding 3 mM L-tryptophan in Landy medium for 72 h at 25 °C. Compared with the control, data that significantly increased (*p* < 0.05) were marked with *

The main genes driving the IAM pathway were *iaam* and *iaah*, and a homologous *iaah* gene was predicted in *P. polymyxa* SC2-M1 by KEGG analysis*.* Therefore, we further cloned an *iaam* gene from *A. tumefaciens* and expressed it by promoters *P*_*gap*_ or *P*_*04420*_ with different strengths to potentially construct the entire IAM pathway in strain SC2-M1. We detected the gene expression levels of *iaam* under promoters *P*_*gap*_ and *P*_*04420*_ in the medium supplemented with L-tryptophan. The results showed that the expression of the *iaam* gene by promoter *P*_*04420*_ was significantly higher than expression by promoter *P*_*gap*_, by 36%. The IAA yield (Fig. 8) of the control strains SC2-M1-P, M1-P_gap_*-*IAM, and M1*-*P_04420_*-*IAM were 13 μg/mL, 14.7 μg/mL, and 17.4 μg/mL, respectively. Compared with the control, the IAA yield of strains M1*-*P_gap_*-*IAM and M1*-*P_04420_*-*IAM was increased by 13% and 34%, respectively. The IAM pathway-expressing strain under promoter *P*_*04420*_ had a higher IAA yield than that under promoter *P*_*gap*_, which was also related to a higher transcription level of *iaam* gene expression under promoter *P*_*04420*_.

#### Overexpression of heterologous IPyA pathways of IAA synthesis in strain SC2-M1

For this section, we selected two heterologous IPyA pathways of IAA synthesis. One IPyA pathway contained genes *aro8*, *kdc*, and *aldH* and was expressed under promoter *P*_*gap*_ or *P*_*04420*_. The genes *aro8* and *kdc* were obtained from yeast, and the gene *aldH* was obtained from *E. coli. E. ludwigii* JP6 was formerly isolated by our group and produces high levels of IAA (data not provided). The other IPyA pathway contained genes *ELJP6_14505*, *ipdC*, and *ELJP6_00725* cloned from the genome of *E. ludwigii* JP6 and expressed only under promoter *P*_*04420*_. The mRNA expression levels of *aro8*, *kdc*, and *aldH* in strain SC2-M1 by promoter *P*_*04420*_ were all higher than those by promoter *P*_*gap*_, and they were increased by 60%, 29%, and 28%, respectively. The genes *aro8*, *kdc*, and *aldH* were expressed at a higher level under promoter *P*_*04420*_. The IAA yield (Fig. 8) of strain M1*-*P_gap_*-*IPyA_AKA_ was increased by 21% compared with the control and reached 15.7 μg/mL. The IAA yield of strain M1*-*P_04420_*-*IPyA_AKA_ was increased by 42% compared with the control and reached 18.4 μg/mL. In general, the IAA yield of strain M1*-*P_04420_*-*IPyA_AKA_ was significantly higher than that of strain M1*-*P_gap_*-*IPyA_AKA_, which was also consistent with the higher transcription levels of genes *aro8*, *kdc*, and *aldH* in strain M1*-*P_04420_*-*IPyA_AKA_ than in strain M1*-*P_gap_*-*IPyA_AKA_. The genes *ELJP6_14505*, *ipdC*, and *ELJP6_00725* from *E. ludwigii* JP6 worked better. The IAA yield of strain M1*-*P_04420_*-*IPyA_JP6_ was significantly increased by 138% compared with the control and reached 31 μg/mL (Fig. 8). Moreover, the transcription levels of these three genes were significantly increased by L-tryptophan by 285%, 222%, and 157%, respectively. The above results show that the IAA yield of strain M1*-*P_04420_*-*IPyA_JP6_ was much higher than that of strains M1*-*P_gap_*-*IPyA_AKA_ and M1*-*P_04420_*-*IPyA_AKA_. The IAA yield of strain M1*-*P_04420_*-*IPyA_JP6_ was also tested by HPLC–MS and the yield of IAA was 32.2 µg/mL, which was almost the same as the result obtained with the colorimetric method (Fig. 8). This also indicated the reliability of the colorimetric method. The heterologous IPyA pathway from *E. ludwigii* JP6 presented the most significant function in strain SC2-M1 in our study, which was stronger than the tested heterologous IAM pathway and the heterologous IPyA pathway containing genes *aro8*, *kdc*, and *aldH*. Through analysis of the different IAM and IPyA pathways, the strongest IPyA pathway from *E. ludwigii* JP6 was finally selected, which could distinctly enhance the IAA synthesis ability of strain SC2-M1.

## Discussion

*P. polymyxa* is commonly found in the soil rhizosphere and can promote plant growth through mechanisms such as producing plant hormones and fixing nitrogen levels [12]. The main plant hormone produced by *P. polymyxa* is IAA [69]. *P. polymyxa* SC2 and its mutant strain SC2-M1 isolated in our laboratory can produce a certain amount of IAA. The genes related to the biosynthesis of IAA in strain SC2-M1 were explored and strengthened to expand the research field of this pathway. In this study, the relevant genes of a native IPyA pathway of IAA synthesis were identified in strain SC2-M1. Two heterogeneous IPyA pathways and a heterogeneous IAM pathway of IAA synthesis were evaluated in strain SC2-M1. To the best of our knowledge, this is the first report of the metabolic engineering of the entire IAA synthesis pathway in *P. polymyxa*.

Studying the functional genes related to IAA biosynthesis requires promoters with appropriate expression strength. In this study, we screened the endogenously and highly expressed genes in strain SC2-M1 through transcriptome data [68], and 22 promoters were cloned for analysis. Our screened promoter sequences were different from those reported previously [60, 65]. The stable promoter *P*_*04420*_ with the strongest expression in our study was obtained and then modified through a synthetic biology strategy. Mutants of promoter *P*_*04420*_ with different expression activities were obtained and analyzed. This study moved further from the irrational selection of the promoter *P*_*LH-77*_ in the early stage of our group’s work [60]. Through the rational strategy screening of this study, the promoter expression system of *P. polymyxa* was further enriched and then used for the identification of genes related to IAA biosynthesis.

The common IAA synthesis pathways in bacteria are mainly the IPyA pathway and the IAM pathway [32, 72]. For the Gram-positive bacterium *P. polymyxa*, the indole-3-pyruvate decarboxylase-encoding gene *ipdC* in the IPyA pathway was previously identified in *P. polymyxa* E681[40]; meanwhile, the *ilvB* gene (*PPYC1_16985*) in the IPyA pathway was detected in *P. polymyxa* YC0136 by our group, and the protein sequence similarity with the *ipdC* gene identified in *P. polymyxa E681* was 100% [18]. As studied in *P. polymyxa* E681 [40], the existence of only an IPyA pathway for IAA biosynthesis was investigated. Based on metabolome analysis, fluorescence quantitative results, and whole-genome protein sequences, this study uncovered three homologous genes related to the IPyA pathway in strain SC2-M1, revealing the existence of the endogenous IPyA pathway. After treatment with L-tryptophan, three genes, *patA* (*PPSC2_17445*), *ilvB3* (*PPSC2_07070*), and *fusE* (*PPSC2_00395*), were significantly induced in strain SC2-M1. In a preliminary study, Shao et al. [32] identified *patB*, *yclC*, and *dhaS* as candidate genes related to IAA synthesis in *B. amyloliquefaciens* SQR9 and constructed a complete IPyA pathway. Using NCBI blast, the genes *patA* and *fusE* of strain SC2-M1 were similar to the genes *patB* and *dhas* of *B. amyloliquefaciens* SQR9 [32], and their protein sequence similarities were 37.6% and 23.8%, respectively. The *ilvB3* gene of strain SC2-M1 was similar to the *ipdC* gene of *P. polymyxa* E681, and their protein sequence similarity was 100% [18]. *PatA*, *ilvB3*, and *fusE* constituted an entire IPyA pathway of IAA synthesis in strain SC2-M1. The strong promoter *P*_*04420*_ was used to overexpress the genes *patA*, *ilvB3*, and *fusE* in *E. coli* and strain SC2-M1 and increased the production of IAA in both strains. The genes *patA*, *ilvB3*, and *fusE* overexpression in strain SC2-M1 significantly increased the IAA yield by 62%, 46%, and 32%, respectively. The IAA synthetic ability of strain SC2-M1 was enhanced, similar to the results of Shao et al. [32] overexpressing *patB*, *yclC*, and *dhaS* in *B. amyloliquefaciens* SQR9 and increasing the IAA production of *B. amyloliquefaciens* SQR9 by 67%, 59%, and 47%, respectively. The co-overexpression of the *patA*, *ilvB3*, and *fusE* genes in strain SC2-M1 could ultimately increase the corresponding IAA yield by 60% which had an obvious effect.

To further enhance the IAA synthetic ability of strain SC2-M1, an attempt was made to construct a heterologous IAM pathway. IAM pathways have rarely been studied in Gram-positive bacteria. As reported, no genes related to the synthesis of the IAM pathway were found in the genome of *B. amyloliquefaciens* SQR9 [32], which was homologous to strain SC2-M1. Through the joint analysis of the genome and metabolome of strain SC2-M1, we found that strain SC2-M1 includes homologous genes *gatA1* (*PPSC2_07840*), *gat* (*PPSC2_12215*), and *yhaA1* (*PPSC2_13350*) related to the gene *iaah* in the IAM pathway but lacks the key tryptophan monooxygenase gene *iaam*. This study attempted to enhance the IAA synthetic ability of strain SC2-M1 by enhancing its IAM pathway, and an *iaam* gene of *A. tumefaciens* was heterologously expressed in strain SC2-M1 with different strength promoters to construct a complete IAM pathway. The results showed that transformant expression of the *iaam* gene could increase the IAA yield of strain SC2-M1 and that the strong promoter *P*_*04420*_ was better than the low-activity promoter *P*_*gap*_, although its increase in IAA was still not ideal. However, the combination and optimization of the IAM pathway in this study laid the foundation for further exploration of the IAM pathway in Gram-positive bacteria.

Meanwhile, two heterologous IPyA pathways were introduced into strain SC2-M1 to enhance IAA synthesis. Three genes, *aro8*, *kdc*, and *aldH*, were cloned and expressed using the promoters *P*_*gap*_ and *P*_*04420*_ to construct entire IPyA pathways, and the IAA yield of the *P*_*04420*_-expressing strain was increased by 42%, up to 18.4 μg/mL. This result also proved the effectiveness of the strong promoter *P*_*04420*_ for the expression of genes in strain SC2-M1. The promoter *P*_*04420*_ was also used to clone three genes, *ELJP6_14505*, *ipdC*, and *ELJP6_00725*, of a predicted IPyA pathway from *E. ludwigii* JP6. The IAA yield of the obtained strain was much higher than that of the original strain, reaching 31 μg/mL. The IAA yield of this IPyA pathway from *E. ludwigii* JP6 was higher than that of the IPyA pathway constructed by *aro8*, *kdc*, and *aldH,* and it was also higher than the endogenous IPyA pathway and the heterologous IAM pathway overexpressing strains. The IPyA pathway from *E. ludwigii* JP6 had good adaptability in strain SC2-M1. During synthesis of IAA through the IPyA pathway, the amounts of some intermediate products, indole-3-ethanol, indole-3-lactic acid, and indole-3-acetamide, were also changed (data not provided). In this study, the use of the novel promoter *P*_*04420*_ to clone the IPyA pathway from *E. ludwigii* JP6 was beneficial for IAA synthesis in *P. polymyxa*, and this could deepen the understanding of the IAA biosynthetic pathway of *P. polymyxa*.

At present, the synthesis of IAA by *P. polymyxa* SC2-M1 has not yet reached an optimal production level, and it is necessary to move further and combine multiple strategies to increase the production of IAA. The genes related to the biosynthesis and regulation of IAA in strain SC2-M1 also need to be further explored and strengthened.

## Conclusions

In this study, a novel, strong, and stable promoter, *P*_*04420*_, was selected, analyzed, and then evaluated to express IAA synthetic genes in *P. polymyxa* SC2-M1*.* Through metabonomic and genomic analysis, native IPyA pathway genes of IAA synthesis in *P. polymyxa* SC2-M1 were predicted. Furthermore, a heterogeneous gene *iaam* in the IAM pathway and two heterogeneous IPyA pathways of IAA synthesis were verified to improve the IAA yield of *P. polymyxa* SC2-M1*.* The genes *ELJP6_14505*, *ipdC*, and *ELJP6_00725* of an entire IPyA pathway from *E. ludwigii* JP6 worked well for significantly increasing the IAA yield from promoter *P*_*04420*_ in *P. polymyxa* SC2-M1. Our results lay the foundation for further optimization of IAA synthetic pathways and the mining regulatory genes in *P. polymyxa* as a framework for future research.

## Materials and methods

### Plasmid and strain construction

The gene fragments of selected promoters were cloned into the *Xba* I and *Bam*H I sites of the plasmid pHY300PLK-gfp-cm using Gibson assembly [73, 74], resulting in plasmid pHY300PLK-Promoter^f^-gfp-cm. The gene fragments of *α-amylase* were cloned from the genome of *P. polymyxa* SC2-M1 and then expressed by promoters *P*_*04420*_, *P*_*04420-4*_, *P*_*04420-6*_, *P*_*04420-8*_, and *P*_*04420-9*_ using Gibson assembly, resulting in the plasmid pHY300PLK-Promoter^g^-amylase. The gene fragments *patA*, *ilvB3*, and *fusE* were cloned from the genome of *P. polymyxa* SC2-M1 and then fused with the promoter *P*_*04420*_, and the resulting segments were cloned into the *Xba* I and *Bam*H I sites of the plasmid pHY300PLK, resulting in plasmids pHY300PLK-patA*,* pHY300PLK-ilvB3, and pHY300PLK-fusE, respectively*.* The genes *patA*, *ilvB3*, and *fusE* were also co-expressed by the promoter *P*_*04420*_ and SD sequence (5′-AGGAGGCATATCAA-3′) in the plasmid pHY300PLK, resulting in the plasmid pHY300PLK-patA-ilvB3-fusE*.* A gene *iaam* was cloned from *A. tumefaciens* and fused with the promoters *P*_*gap*_ or *P*_*04420*_ and then cloned into the *Xba* I and *Bam*H I sites of the plasmid pHY300PLK, resulting in the plasmids pHY300PLK-P_gap_-IAM and pHY300PLK-P_04420_-IAM, respectively. The genes *aro8* and *kdc* from *yeast,* and the gene *aldH* from *E. coli* [30] were synthesized (Beijing Genomics Institution, China) and then co-expressed by promoters *P*_*gap*_ or *P*_*04420*_ in the plasmid pHY300PLK, resulting in the plasmids pHY300PLK-P_gap_-IPyA_AKA_ and pHY300PLK-P_04420_-IPyA_AKA_, respectively. The gene fragments *ELJP6_14505*, *ipdC*, and *ELJP6_00725* were cloned from the genome of *Enterobacter ludwigii* JP6 (NCBI Reference Sequence: NZ_CP040256.1) and then cloned into the *Xba* I and *Bam*H I sites of the plasmid pHY300PLK and co-expressed by promoter *P*_*04420*_ and SD sequence (5′-AGGAGGCATATCAA-3′), resulting in the plasmid pHY300PLK-P_04420_-IPyA_JP6_. The gene fragments of promoters *P*_*gap*_ and *P*_*04420*_ were cloned into the plasmid pHY300PLK, resulting in the plasmids pHY300PLK-P_gap_ and pHY300PLK-P_04420_, respectively.

*E. coli* DH5α was used for plasmid subcloning and amplification. *E. coli* DH5α, *P. polymyxa* SC2-M1, and *B. subtilis* 168 were selected as the promoter-expressing strains. *E. coli* DH5α and *P. polymyxa* SC2-M1 were selected as the α-amylase-expressing and IAA pathway-expressing strains. The corresponding transformation methods of *P. polymyxa* SC2-M1 were conducted as previously reported by our group [60]. The strains and plasmids used in this study are listed in Table 2. The corresponding primers are summarized in Additional file 1: Table S1.

**Table 2:** Strains and plasmids used in this study

Strains or plasmids	Genotype/properties	Source/references
Strain		
*E. coli* DH5α	*F-φ80 lac ZΔM15 Δ(lacZYA-arg F) U169 endA1 recA1 hsdR17*(*rk −*, *mk* +)* supE44λ- thi-1 gyrA96 relA1 phoA*	TransGen Biotech
CQDH5α-Qiu	*E. coli* DH5α derivative; {pHY300PLK-*gfp-cm-ter*}	[60]
CQDH5α-promoter^a^	*E. coli* DH5α derivative; {pHY300PLK-*Promoter-gfp-cm-ter*}	This work
CQDH5α-amy^b^	*E. coli* DH5α derivative; {pHY300PLK-*P*_*04420*_*/P*_*04420-4*_*/P*_*04420-6*_*/P*_*04420-8*_*/P*_*04420-9*_*-α-amylase-ter*}	This work
DH5α-patA	DH5α derivative; {pHY300PLK*-P*_*04420*_*-patA*}	This work
DH5α-ilvB	DH5α derivative; {pHY300PLK*-P*_*04420*_*-ilvB3*}	This work
DH5α-fusE	DH5α derivative; {pHY300PLK*-P*_*04420*_*-fusE*}	This work
DH5α-C	DH5α derivative; {pHY300PLK*-P*_*04420*_*-patA-ilvB3-fusE*}	This work
DH5α-P_gap_-IAM	DH5α derivative; {pHY300PLK*-P*_*gap*_*-iaam*}	This work
DH5α-P_04420_-IAM	DH5α derivative; {pHY300PLK*-P*_*04420*_*-iaam*}	This work
DH5α-P_gap_-IPyA_AKA_	DH5α derivative; {pHY300PLK*-P*_*gap*_*-aro8-kdc-aldH*}	This work
DH5α-P_04420_-IPyA_AKA_	DH5α derivative; {pHY300PLK*-P*_*04420*_*-aro8-kdc-aldH*}	This work
DH5α-P_04420_-IPyA_JP6_	DH5α derivative; {pHY300PLK*-P*_*04420*_*-ELJP6_14505-ipdC-ELJP6_00725*}	This work
*B. subtilis* 168	*trpC2*	BGSC, [60]
CQ168-Qiu	*B. subtilis* 168 derivative; {pHY300PLK*-gfp-cm-ter*}	[60]
CQ168-promoter^c^	*B. subtilis* 168 derivative; {pHY300PLK*-Promoter-gfp-cm-ter*}	This work
*P. polymyxa* SC2	Wild type, isolated from the rhizosphere of pepper plants in Guizhou, China	[66]
*P. polymyxa* SC2-M1	Spontaneous mutant of *P. polymyxa SC2*	[68]
CQM1-Qiu	*P. polymyxa* SC2-M1 derivative; {pHY300PLK*-gfp-cm-ter*}	[60]
CQM1-Promoter^d^	*P. polymyxa* SC2-M1 derivative; {pHY300PLK*-Promoter-gfp-cm-ter*}	This work
CQM1-amy^e^	*P. polymyxa* SC2-M1 derivative; {pHY300PLK*-P*_*04420*_*/P*_*04420-4*_*/P*_*04420-6*_*/P*_*04420-8*_*/P*_*04420-9*_*-*α-amylase-ter}	This work
SC2-M1-P	SC2-M1derivative; {pHY300PLK}	This work
M1-patA	SC2-M1derivative; {pHY300PLK*-P*_*04420*_*-patA*}	This work
M1-ilvB	SC2-M1derivative; {pHY300PLK*-P*_*04420*_*-ilvB3*}	This work
M1-fusE	SC2-M1derivative; {pHY300PLK*-P*_*04420*_*-fusE*}	This work
M1-C1	SC2-M1 derivative; {pHY300PLK*-P*_*04420*_*-patA–ilvB3–fusE*}	This work
M1-P_gap_-IAM	SC2-M1 derivative; {pHY300PLK*-P*_*gap*_*-iaam*}	This work
M1-P_04420_-IAM	SC2-M1 derivative; {pHY300PLK*-P*_*04420*_*-iaam*}	This work
M1-P_gap_-IPyA_AKA_	SC2-M1 derivative; {pHY300PLK*-P*_*gap*_*-aro8-kdc-aldH*}	This work
M1-P_04420_-IPyA_AKA_	SC2-M1 derivative; {pHY300PLK*-P*_*04420*_*-aro8-kdc-aldH*}	This work
M1-P_04420_-IPyA_JP6_	SC2-M1 derivative; {pHY300PLK*-P*_*04420*_*-ELJP6_14505-ipdC-ELJP6_00725*}	This work
Plasmid		
pHY300PLK	*E. coli* and *B. subtilis* shuttle vector; Amp^r^, Tet^r^	TaKaRa
pHY300PLK-gfp-cm	pHY300PLK-*gfp-cm-ter*	[60]
pHY300PLK-Promoter^f^-gfp-cm	pHY300PLK*-Promoter-gfp-cm-ter*	This work
pHY300PLK-Promoter^g^-amylase	pHY300PLK*-P*_*04420*_*/P*_*04420-4*_*/P*_*04420-6*_*/P*_*04420-8*_*/P*_*04420-9*_*-*α-amylase-ter	This work
pHY300PLK-P_gap_	pHY300PLK*-P*_*gap*_	[60]
pHY300PLK-P_04420_	pHY300PLK*-P*_*04420*_	This work
pHY300PLK-patA	pHY300PLK*-P*_*04420*_*-patA*	This work
pHY300PLK-ilvB3	pHY300PLK*-P*_*04420*_*-ilvB3*	This work
pHY300PLK-fusE	pHY300PLK*-P*_*04420*_*-fusE*	This work
pHY300PLK-patA-ilvB3-fusE	pHY300PLK*K-P*_*04420*_*-patA-ilvB3-fusE*	This work
pHY300PLK-P_gap_-IAM	pHY300PLK*K-P*_*gap*_*-iaam*	This work
pHY300PLK-P_04420_-IAM	pHY300PLK*-P*_*04420*_*-iaam*	This work
pHY300PLK-P_gap_-IPyA_AKA_	pHY300PLK*-P*_*gap*_*-aro8-kdc-aldH*	This work
pHY300PLK-P_04420_-IPyA_AKA_	pHY300PLK*-P*_*04420*_*-aro8-kdc-aldH*	This work
pHY300PLK-P_04420_-IPyA_JP6_	pHY300PLK*-P*_*04420*_*-ELJP6_14505-ipdC-ELJP6_00725*	This work

^a^The *E. coli* DH5α-based strains containing all the promoter fragments in plasmid pHY300PLK-gfp-cm in this work

^b^The *E. coli* DH5α-based strains expressing *α-amylase* by promoter *P*_*04420*_ and its derivatives in this work

^c^The *B. subtilis* 168-based strains containing all the promoter fragments in plasmid pHY300PLK-gfp-cm in this work

^d^The *P. polymyxa* SC2-M1-based strains containing all the promoter fragments in plasmid pHY300PLK-gfp-cm in this work

^e^The *P. polymyxa* SC2-M1-based strains expressing *α-amylase* by promoter *P*_*04420*_ and its derivatives in this work

^f^Containing all the promoter fragments in this work

^g^Containing promoter fragments of *P*_*04420*_ and its derivatives in this work

### Medium and batch cultivation of strains

Cultivation of strains *E. coli* DH5α, *P. polymyxa* SC2, *P. polymyxa* SC2-M1, and *B. subtilis* 168 was performed with liquid Luria–Bertani (LB) medium supplemented with 30 μg/mL ampicillin, 15 μg/mL or 30 μg/mL tetracycline, or nothing [75]. For solid culture, 20 g/L agar was added. For IAA production of *P. polymyxa*, the bacteria were grown for 72 h in Landy medium containing 3 mM L-tryptophan at 25 °C and 90 rpm [18]. For IAA production of *E. coli*, R2A medium was used [76]. All strains were cultivated in triangular flasks for batch cultivation. Single colonies of fresh strains were preincubated and then transferred to fresh media for growth curve testing. The values of culture optical density (OD_600_) were tested with a BioPhotometer Plus (Eppendorf, Germany) to construct growth curves.

### Metabolomic analysis

Strain SC2-M1 was inoculated into 50 mL liquid LB and then cultured overnight at 37 °C as a seed solution. The next day, strain SC2-M1 was diluted to OD_600_ = 0.5 and inoculated into 50 mL Landy medium at an inoculation amount of 1%. The Landy medium of the test group (M1-T) contained 3 mM L-tryptophan compared with the control group (M1) using only Landy medium. Each group contained 4 biological replicates that were cultured at 37 °C for 9 h. For each biological replicate, a total of 1.5 mL of cell solution was taken, flash-frozen with liquid nitrogen, and then sent to Beijing Genomics Institution (BGI, China) for metabolomic analysis.

### Quantitative real-time PCR

All measurements were independently conducted in the manner previously reported by our group [68].

### Measurements of the whole-cell fluorescence intensities

Single colonies of fresh strains were preincubated in LB liquid medium for 24 h and then transferred to fresh media at a final concentration of 10% for 24 h incubation, or for growth curve testing. The cultivated cells were washed and then diluted with 50 mmol/L phosphate-buffered saline (PBS, pH 7) to analyze the whole-cell fluorescence intensities [60] on a CLARIO star, a multimode microplate reader (BMG LABTECH, Germany). The growth time of all recombinant bacteria was 24 h, and the gain value was 1600. To evaluate the strength of different promoters, the values of the whole-cell relative fluorescence units were divided by the cell density OD_600_ (RFU/OD_600_) [70]. The epifluorescence images of the strains were taken by a fluorescence microscope (Zeiss, Germany). The laser intensity was 25, the exposure value was 85, and the gain value was 100.

### Assay method of α-amylase activities by starch hydrolysis on plates

An appropriate amount of strains cultured overnight was adjusted to OD_600_ = 0.5 with sterile LB, and then 0.3 μL was placed onto LB solid medium containing 1% starch to cultivate in an incubator. Several hours later, an appropriate amount of I_2_–KI solution was placed on the petri dish. After 10 min in the dark, the petri dish was re to measure the size of the transparent circle produced by the bacterial extracellular α-amylase by hydrolyzing the surrounding starch. The activity of α-amylase was proportional to the size of the transparent circle [77].

### Analytical methods of IAA and related derivatives

#### Colorimetric method

This method was performed as previously reported by our group [18].

#### HPLC–MS analysis

The concentration of IAA was quantified by HPLC–MS according to a reported procedure [32].

## Supplementary Information

**Additional file 1: Table S1.** The DNA oligos used in this study. **Table S2.** Enrichment results of metabolic pathways. **Table S3.** The predicted characteristics of selected promoters. **Table S4.** The sequence characteristics of original* P*_*04420*_ and its modified derivatives. **Figure S1.** Schematic representation of conserved bases (-10, -35, and SD regions) in the promoter sequences of 77 high transcription level genes. **Figure S2.** Fluorescent microscopic observation of GFP that expressed by high strength promoters in strain SC2-M1. **Figure S3.** Fluorescence intensity of GFP that expressed by different promoters. **Figure S4.** Assay of α-amylase activities by starch hydrolysis on plates.

## Data Availability

All data of this study are included in the published article and its supplemental files.

## Abbreviations

PGPR: Plant growth-promoting rhizobacteria
IAA: Indole-3-acetic acid
IPyA: Indole-3-pyruvic acid
IAM: Indole-3-acetamide
IAN: Indole-3-acetonitrile
TAM: Tryptamine
TSO: Tryptophan side-chain oxidase
IAAlD: Indole-3-acetaldehyde
IPDC: Indole-3-pyruvate decarboxylase
IAOx: Indole-3-acetaldoxime
AEC: Auxin efflux carrier
RPKM: Reads per kilobase of transcript per million reads mapped
RBS: Ribosome binding site
GFP: Green fluorescent protein
