# Supplementary material for: Identification and combinatorial engineering of indole-3-acetic acid synthetic pathways in Paenibacillus polymyxa
Source: Biotechnol Biofuels Bioprod. 2022 Aug 11;15:81. doi: 10.1186/s13068-022-02181-3 (PMC9367139; doi:10.1186/s13068-022-02181-3)
Supplement: Supplementary file 1 — Additional file 1: Table S1. The DNA oligos used in this study. Table S2. Enrichment results of metabolic pathways. Table S3. The predicted characteristics of selected promoters. Table S4. The sequence characteristics of original P04420 and its modified derivatives. Figure S1. Schematic representation of conserved bases (-10, -35, and SD regions) in the promoter sequences of 77 high transcription level genes. Figure S2. Fluorescent microscopic observation of GFP that expressed by high strength promoters in strain SC2-M1. Figure S3. Fluorescence intensity of GFP that expressed by different promoters. Figure S4. Assay of α-amylase activities by starch hydrolysis on plates. [file 13068_2022_2181_MOESM1_ESM.docx]

**Table S1** The DNA oligos used in this study

| Primer | Sequence (5’-3’) | Purpose |
| --- | --- | --- |
| SC2-F | GTCGTCAATAACATTTGGCAGGTCTA | Verifying and sequencing of strain SC2 |
| SC2-R | TGCGGCAAGAGCGGTTAGAGCGAGT |  |
| P_25430_-F | CTAGTCTAGAGCTGTTCTCCGTCATTTCTAC | Cloning promoter *P_25430_* |
| P_25430_-R | CGCGGATCCCAGTCGTTACACCTCCTTTC |  |
| P_15240_-F | CTAGTCTAGATAGTATGAAGAGGCTTGTAACA | Cloning promoter *P_15240_* |
| P_15240_-R | CGCGGATCCGTAAATCCCTCCAAATTGTTTG |  |
| P_22955_-F | CTAGTCTAGAGGATTAACTTCCTACTTTCCATC | Cloning promoter *P_22955_* |
| P_22955_-R | CGCGGATCCAGGGCACGCCTCCTTTG |  |
| P_13385_-F | CTAGTCTAGATTCGGAGTAGCACAACCTGAT | Cloning promoter *P_13385_* |
| P_13385_-R | CGCGGATCCCCTCGTAATCTCCTTTCTCTG |  |
| P_04420_-F | CTAGTCTAGAGTTCATCCCTCCACATCG | Cloning original promoter *P_04420_* |
| P_04420_-R | CGCGGATCCTCAAATCACTCCTCTTTCTTAATC |  |
| P_05390_-F | CTAGTCTAGATCTTGACTATTTGGAGATCATC | Cloning promoter *P_05390_* |
| P_05390_-R | CGCGGATCCCTGAAAGGGAACCGGAAATG |  |
| P_09115_-F | GCTCTAGAATAGTAACAATTCGAAACTCCGG | Cloning promoter *P_09115_* |
| P_09115_-R | ACGCGTCGACGTTGCTGCCTCCTCTCCCAAAC |  |
| P_22445_-F | CTAGTCTAGATTCCGAAGAGCATTGCTGAAG | Cloning promoter *P_22445_* |
| P_22445_-R | CGCGGATCCTTGAACAATTCCTCCTCAATAAC |  |
| P_21510_-F | CTAGTCTAGAAAGAACGCGATGACTGGTGGG | Cloning promoter *P_21510_* |
| P_21510_-R | CGCGGATCCTATACAGACCCCCTTATTGCCGC |  |
| P_23030_-F | CTAGTCTAGATGCGCAAGATGATAAATAAAAACAAC | Cloning promoter *P_23030_* |
| P_23030_-R | CGCGGATCCTTTAAATTTCCTCCCTGAAGGTAAATG |  |
| P_10125_-F | CTAGTCTAGAACATAAGACAGGCGCTGTGTTATACTG | Cloning promoter *P_10125_* |
| P_10125_-R | CGCGGATCCCCTGTTCACCTCCTTCATTATAATCGC |  |
| P_22340_-F | CTAGTCTAGAGCATCATCCGTAAAGAAGGTAAAGC | Cloning promoter *P_22340_* |
| P_22340_-R | CGCGGATCCCTCAAGTTTTCACTCCCTTCAG |  |
| P_15565_-F | CTAGTCTAGAAGAATATTCCTTAGGGAATGTTCTC | Cloning promoter *P_15565_* |
| P_15565_-R | CGCGGATCCTACCCATTGCTTTCTCCAATGC |  |
| P_00160_-F | CTAGTCTAGACAAATGTGCCTGGGTATATCTGAAC | Cloning promoter *P_00160_* |
| P_00160_-R | CGCGGATCCTGCCTAACGACCACCTCTTTCCT |  |
| P_25430_-F | GCTCTAGAACGGTTGTGCCTCCTTAGATAATG | Cloning promoter *P_25430_* |
| P_25430_-R | CGCGGATCCTCTTTACACCTCCTTATGGACATAAGG |  |
| P_22450_-F | CTAGTCTAGATGCGGGATTGAAGTGGGAGC | Cloning promoter *P_22450_* |
| P_22450_-R | CGCGGATCCGTTGGTTGCCACCCCCTTCC |  |
| P_16895_-F | CTAGTCTAGATGACAGGTATTTCGGACTCGC | Cloning promoter *P_16895_* |
| P_16895_-R | CGCGGATCCCAGTTTTCCCTCCCTCCGA |  |
| P_22370_-F | CTAGTCTAGAACATCAAACGTGGTGGTAAGG | Cloning promoter *P_22370_* |
| P_22370_-R | CGCGGATCCTGCTTCACCACCCAATTCTTC |  |
| P_00970_-F | CTAGTCTAGATTCTGACTGTTGGCGACAAACAAC | Cloning promoter *P_00970_* |
| P_00970_-R | CGCGGATCCCTCCATATTCCTCCGCACCCT |  |
| P_19355_-F | CTAG TCTAGAGACTCTCGTGTAGAACGTGCG | Cloning promoter *P_19355_* |
| P_19355_-R | CGC GGATCCTTGCTTGCACCTCCTTATGTC |  |
| P_22425_-F | CTAGTCTAGATCATCGTATTGGACGCTCTTAC | Cloning promoter *P_22425_* |
| P_22425_-R | CGCGGATCCTACGCGAGCACCTCCTCTACCT |  |
| P_spo0A_-F | CTAGTCTAGAGGAATAACTCAGAATTGCAAG | Cloning promoter *P_spo0A_* |
| P_Spo0A_-R | CGCGGATCCTGTACTTCCTCCTTATTGAGTG |  |
| P_04420_-InF-F | GCCCAAGCTTCTAGAGTTCATCCCTCCACATCGTT | Cloning promoter *P_04420_* without *Bam*H I |
| P_04420-_InF-R | TTCTCCTTTACTCATTCAAATCACTCCTCTTTCTTA |  |
| p300-fx-F | ATGAGTAAAGGAGAAGAACTTTTC | Linearizing pHY300PLK by PCR |
| p300-fx-R | TCTAGAAGCTTGGGCAAAGCGT |  |
| 04-BN20-F | GCCCAAGCTTCTAGAGTTCATCCCTCCACATCGT | Cloning promoter *P_04420_* by adding *Nhe* I |
| 04-BN20-R | TTTACTCATGCTAGCGGATCCTCAAATCACTCCTCT |  |
| fx300-04BN20-F | GCTAGCATGAGTAAAGGAGAAGAAC | Linearizing pHY300PLK |
| fx300-04BN20-R | TCTAGAAGCTTGGGCAAAGCCG |  |
| 04-4-amy-F | GTGATTTGAGGATCCATGAAAAGGAACCATACGATGATGC | Amplifing α-amylase gene |
| 04-4-amy-R | CGGCATTATCTCATATCAAGCACTCGCGGTATCC |  |
| fx300-04-4-amy-F | TATGAGATAATGCCGACTGTACT | Linearizing pHY300PLK and amplifing α-amylase gene |
| fx300-04-4-amy-R | GGATCCTCAAATCACTCCTCT |  |
| fx300-04-6-amy-R | GGATCCTCAAATCACCTCCT |  |
| 04-8-amy-F | GAGGAGTGAGGATCCATGAAAAGGAACCATACGATGATGC |  |
| fx300-04-8-amy-R | GGATCCTCACTCCTCTTTCTT |  |
| 04-9-amy-F | AAAGAGGAGGGATCCATGAAAAGGAACCATACGATGATGC |  |
| fx300-04-9-amy-R | GGATCCCTCCTCTTTCTTAATCT |  |
| Amy-R | TAGAGTATCCACCAGCCATTTG | Verifying the α-amylase gene |
| GAP-F | GCTCTAGACGAATCGTTCGGCTATTA | Cloning promoter *P_gap_ for* expressing gene *iaam* |
| GAP-R | AGGTGAAGCTGACATGGATCCTGAATAGTTCCTCCTAGATTTCG |  |
| 04420-F | GCTCTAGAGTTCATCCCTCCACATC | Cloning promoter *P_04420_ for* expressing gene *iaam* |
| 04420-R | AGGTGAAGCTGACATGGATCCTCAAATCACTCCTCTTTC |  |
| GFP-R | CTCGCAAAGCATTGAACACC | Sequencing of *gfp* sequence |
| GAP-IAM-F | CGAAATCTAGGAGGAACTATTCAGGATCCATGTCAGCTTCACCT | Cloning gene *iaam* and expressed by promoter *P_gap_* |
| GAP-IAM-R | TGATCACCACGCCGAAACAAGCGC |  |
| 04420-IAM-F | GAAAGAGGAGTGATTTGAGGATCCATGTCAGCTTCACCT | Cloning gene *iaam* and expressed by promoter *P_04420_* |
| 04420-IAM-R | TGATCACCACGCCGAAACAAGCGC |  |
| GAP-F1 | CGTCTAGACGAATCGTTCGGCTATTA | Cloning promoter *P_gap_* to express IPyA pathway genes |
| GAP-R1 | GTCTCTTGATTCAGGTAAAGTCATTGAATAGTTCCTCCTAGATTTCG |  |
| aro8-F | CGAAATCTAGGAGGAACTATTCAATGACTTTACCTGAATCAAGAGAC | Cloning gene *aro8* |
| aro8-R | CAATTGTAACAGGTGCCATCTATTTGGAAATACCAAATTCTTCG |  |
| kdc-F | CGAAGAATTTGGTATTTCCAAATAGATGGCACCTGTTACAATTG | Cloning gene *kdc* |
| kdC-R | CTGGATAAGCCTGGAGGCCTGACTATTTTTTATTTCTTTTAAGTGC |  |
| aldH-F | GCACTTAAAAGAAATAAAAAATAGTCAGGCCTCCAGGCTTATCCAG | Cloning gene *aldH* |
| aldH-R | AGTACAGTCGGCATTATCTCATAATGAATTTTCATCATCTGGC |  |
| 04420-F2 | GCCCAAGCTTCTAGAGTTCATCCCTCCACATCGTT | Cloning promoter *P_04420_* to express IPyA pathway genes |
| 04420-R2 | TTCAGGTAAAGTCATTCAAATCACTCCTCTTTCTTAATCT |  |
| 04420-F3 | CGTCTAGA GTTCATCCCTCCACATC |  |
| 04420-R3 | CGGACTTTATCCGTCATCATTCAAATCACTCCTCTTTC |  |
| 04420-R4 | GGAATTTGTGCACTCATTCAAATCACTCCTCTTTC |  |
| 04420-R5 | CTGGATTTATACTCATTCAAATCACTCCTCTTTC |  |
| patA-F | GAGTGATTTGAATGGAGCATTTGCTTAAC | Cloning gene *patA* |
| patA-R | GTGCACTCATTTGATATGCCTCCTTAACTCCTAACAATTCCCC |  |
| ilvB3-F | GGCATATCAAATGAGTGCACAAATTCCTG | Cloning gene *ilvB3* |
| ilvB3-R | GATTTATACTCATTTGATATGCCTCCTTACTCGTCCCCCATCAGC |  |
| fusE-F | GGCATATCAAATGAGTATAAATCCAGAAC | Cloning gene *fusE* |
| fusE-R | GGAATTCTCATACCAAACTCCTCTTTTC |  |
| pHY300PLK-F | GTCAGATTTCGTGATGCTTGTC | Verifying and sequencing the plasmid pHY300PLK |
| pHY300PLK-R | GGATCAACTTTGGGAGAGAGTTC |  |
| 04420An-F | CGTCTAGAGTTCATCCCTCCACATC | Cloning promoter *P_04420_* for the amplification of IPyA pathway (JP6) |
| 04420An-R | CGGACTTTATCCGTCATCATTCAAATCACTCCTCTTTC |  |
| An-Ipdc--F | GAAAGAGGAGTGATTTGAATGATGACGGATAAAGTCCG | Cloning gene *ELJP6_14505* |
| AnR-R | GCAGTATGGGGTTCGCATTTGATATGCCTCCTTTACGCCGGGCGAACG |  |
| IpdcR-F | CGTTCGCCCGGCGTAAAGGAGGCATATCAAATGCGAACCCCATACTGC | Cloning gene *ipdC* |
| IPDCR-R | GGGAGGATTGTTTGTCATTTGATATGCCTCCTTCAGGCGTTATTACGCGC |  |
| QR-F | GCGCGTAATAACGCCTGAAGGAGGCATATCAAATGACAAACAATCCTCCC | Cloning gene *ELJP6_00725* |
| Q-R | CGGGATCCTTAGAACAGCCCCAGTGG |  |
| aro8-F1 | TCTTGAAGCCTTACTTGAGT | Identification of gene *aro8* expression at transcription level |
| aro8-R1 | GCATCAGATTGTGGTAGATAC |  |
| kdC-F2 | ATTCTCTGTTCGTGATTTCG | Identification of gene *kdC* expression at transcription level |
| kdC-R2 | ATCTTGGCTTATAGTCCTCTT |  |
| aldH-F3 | CGGTCCATAGCTTTATTCGGCCTGTTCTTCTGATGTG | Identification of gene *aldH* expression at transcription level |
| aldH-R3 | GCCTGTTCTTCTGATGTG |  |
| gatA-F | TAGCGAAATACGGATTGGA | Identification of gene *gatA* expression at transcription level |
| gatA-R | ATACGAATTAGCATAGGTTCAC |  |
| patA-F | TGAGGGAGCCTTCTATCT | Identification of gene *patA* expression at transcription level |
| patA-R | CTGTCCGTAAGCGTAAGA |  |
| alaT-F | CTGGCGTATTGGATATACTTG | Identification of gene *alaT* expression at transcription level |
| alaT-R | GATTATAGGCTTCTACCATTCG |  |
| ilvB3-F | AATCGTAATCATCAACAACCA | Identification of gene *ilvB3* expression at transcription level |
| ilvB3-R | CGTGCTTCTTCCTTATTCG |  |
| fusE-F | TGGATCAGAACTTGGAGATTA | Identification of gene *fusE* expression at transcription level |
| fusE-R | TTCGTAGAACATCAGTCAGA |  |
| sdr2-F | AACATACAGCCGTAGAGG | Identification of gene *sdr2* expression at transcription level |
| sdr2-R | TGATGACTTCATTGACAACTC |  |
| PPSC2_05390-F | GTTCTTCAAACACAAGTCATTC | Identification of gene *PPSC2_05390* expression at transcription level |
| PPSC2_05390-R | TTTGGAGGGATAACCAGATAA |  |
| niT2-F | ATGTATGTCATCGCTTGTAAC | Identification of gene *niT2* expression at transcription level |
| niT2-R | GAACCAAGGCTGATTCAAT |  |
| ELJP6_14505-F | ATGATGACGGATAAAGTCCG | Identification of gene *ELJP6_14505* expression at transcription level |
| ELJP6_14505-R | CGAAATAGTAGGTCAGCGC |  |
| ipdc-F | ATGCGAACCCCATACTGC | Identification of gene *ipdC* expression at transcription level |
| ipdc-R | GCTCATCGCCAGTTTGTTC |  |
| ELJP6_00725-F | CAATCCTCCCTCATCGCG | Identification of gene *ELJP6_00725* expression at transcription level |
| ELJP6_00725-R | GAAGGTCGCCAATGACTTC |  |

**Table S2** Enrichment results of metabolic pathways

| Pathway | Count | Pathway ID | KEGG Names |  |
| --- | --- | --- | --- | --- |
| Positive ion mode |  |  |  |  |
| Tryptophan metabolism | 17 | map00380 | Alpha-ketoadipicacid; Indole-3-acetamide; N-formylkynurenine; Indole-3-pyrubate; 5-hydroxyindoleacetate; Serotonin; 8-methoxykynurenicacid; L-tryptophan; Indole; Tryptamine; Kynurenicacid; Questiomycina; N-[(5-hydroxy-1h-indol-3-y) acetyl]glycine; 5-methoxy-3-indoleaceate; Indole-3-acetic acid; Indole-3-ethanol; Indole-3-acetaldehyde |  |
| Metabolic pathways | 41 | map01100 | Spermidine; L-serineo-phosphate; N-acetyl-d-mannosamine; Pyridoxal; O-succinyl-l-homoserine; VanillylacidSuccinicacid; N-acetyl-l-asparticacid; D-pantothenicacid; Alpha-ketoadipicacid; 3-phosphonooxypyruvicd; Pyroglutamate; L-allo-threonine; N-acetyl-l-glutamate; N-formylkynurenine; 3-methoxytyramine; Indole-3-pyrubate; 5-hydroxyindoleacetate; Serotonin; L-tryptophan;Indole; (e)-p-coumaricacid; Tryptamine; Kynurenicacid; 1,2-dihydroxy-3-keto-5-methylthiopentene; Naphthalene; Levodopa; Flavinmononucleotide; Biotin; Atrazine; Estriol; Trans-cinnamate; TOLuene; Indole-3-aceticacid; Dopamine; Indole-3-acetaldehyde; Methyl(2r,3s)-3-hydroxy-8-methyl-8-azabicyclo[3.2.1]octane-2-carboxylate; 2-amino-1,3,4-octadecanetriol; S-adenosylmethionine |  |
| Tyrosine metabolism | 8 | map00350 | 4-hydroxyphenylaceticacid; Tyramine; DopamineAdenylthiomethylpentose；Succinic acid; Vanillyl 3-methoxytyramine; (e)-p-coumaricacid; Levodopa; mandelic acid; |  |
| Microbial metabolism in diverse environments | 22 | map01120 | L-serineo-phosphate; Pyridoxal; Alpha-ketoglutaric acid; Succinic acid; Alpha-ketoadipic acid; 3-phosphonooxypyruvic acid; Glutaric acid; Cis-acetylacrylicacid; Aniline; 1-methoxypyrene; 6-hydroxypseudooxynicotine; Pyrene; Naphthalene; Acetophenone; Benzaldehyde; Atrazine; S-formylglutathione; 4-hydroxyphenylacetic acid; Trans-cinnamate; Benzene; TOLuene; 2,4-diamino TOL |  |
| Glycine, serine and threonine metabolism | 4 | map00260 | 1. serine o-phosphate; 3-phosphonooxypyruvic acid; Llo-al-threonine; L-tryptophan |  |
| Phenylalanine  metabolism | 6 | map00360 | 1. Succinic acid; (e)-p-coumaric acid; 4-hydroxyphenylaceticacid; Trans-cinnamate; Hippuric; N-acetyl-l-phenylalanine |  |
| Methane metabolism | 5 | map00680 | L-serine o-phosphate; Alpha-ketoadipic acid; 3-phosphonooxypyruvic acid; S-formylglutathione; Tyramine |  |
| Biosynthesis  amino acids | 8 | map01230 | L-serineo-phosphate; O-succinyl-l-homoserine; Alpha-ketoglutaric acid; Alpha-ketoadipic acid; 3-phosphonooxypyruvic acid; N-acetyl-l-glutamate; L-tryptophan; S-adenosylmethionine |  |
| Degradation of aromatic compounds | 9 | map01220 | Succinic acid; (e)-p-coumaric acid; Naphthalene; Acetophenone; Benzaldehyde; 4-hydroxyphenylaceticacid; Trans-cinnamate; Benzene; TOLuene |  |
| Lysine degradation | 4 | map00310 | Alpha-ketoglutaric acid; Succinic acid; Alpha-ketoadipic acid; Glutaric acid |  |
| Oxidative phosphorylation | 3 | map00190 | Phosphoric acid; Succinic acid; Flavin mononucleotide |  |
| Alanine, aspartate and glutamate metabolism | 3 | map00250 | Alpha-ketoglutaric acid; Succinic acid; N-acetyl-l-aspartic acid |  |
| Cysteine and methioninemetabolism | 4 | map00270 | L-serineo-phosphate; O-succinyl-l-homoserine; 1,2-dihydroxy-3-keto-5-methylthiopentene; S-adenosylmethionine |  |
| Citrate cycle | 2 | map00020 | Alpha-ketoglutaric acid; Succinic acid |  |
| Arginine biosynthesis | 2 | map00220 | Alpha-ketoglutaric acid; N-acetyl-l-glutamate |  |
| beta-Alaninemetabolim | 2 | map00410 | Arginine biosynthesis Spermidine; D-pantothenic acid |  |
| Biosynthesis of secondary metabolites | 22 | map01110 | O-succinyl-l-homoserine; Alpha-ketoglutaric acid; Succinic acid; D-pantothenic acid; N-acetyl-l-glutamate; Acetone cyanohydrin; (-)-physostigmine; L-tryptophan; Indole; Tryptamine; Levodopa; Flavin mononucleotide; 2-oxindole; Cinnamyl alcohol; Trans-cinnamate; Tyramine; Dopamine; Methyl(2r,3s)-3-hydroxy-8-methyl-8-azabicyclo[3.2.1]octane-2-carboxylate; 7-hydroxycoumarine; Sparteine; S-adenosylmethionine |  |
| Carbon metabolism | 4 | map01200 | L-serine o-phosphate; Alpha-ketoglutaric acid; Succinic acid; 3-phosphonooxypyruvic acid |  |
| Negative ion mode |  |  |  |  |
| Tryptophan metabolism | 11 | map00380 | N-formylkynurenine; 8-methoxykynurenic acid; Tryptamine; L-tryptophan; N-[(5-hydroxy-1h-indol-3-yl)acetyl]glycine;Kynurenic acid; Indole-3-acetamide; 2-amino-3-methoxybenzoic acid; 5-hydroxyindole-3-acetic acid; Indole-3-acetic acid; Indole-3-pyruvic acid |  |
| Biosynthesis  of amino acids | 5 | map01230 | L-2-succinylamino-6-oxoheptanedioic acid; Citrate; L-serineo-phosphate; L-tryptophan; Phenylpyruvic acid |  |
| Oxidative phosphorylation | 2 | map00190 | Pyrophosphoric acid; Phosphoric acid |  |
| Tyrosine metabolism | 3 | map00350 | Vanillyl mandelic acid; L-dopa; Homovanillic acid |  |
| ABC transporters | 3 | map02100 | Sulfuric acid; Sucrose; Phosphoric acid |  |
| Quorum sensing | 2 | map02024 | Sulfuric acid; Phosphoric acid |  |
| Glycine, serine and threonine metabolism | 2 | map00260 | L-serine o-phosphate; L-tryptophan |  |
| Aminoacyl-tRNA biosynthesis | 2 | map00970 | L-serine o-phosphate; L-tryptophan |  |
| Two-component system | 2 | map00220 | Citrate; Phosphoric acid |  |
| Phenylalanine, tyrosine and tryptophan biosynthesis | 2 | map00400 | L-tryptophan; Phenylpyruvic acid |  |
| Cysteine and methionine metabolism | 2 | map00270 | Sulfuric acid; L-serine o-phosphate |  |
| 2-Oxocarboxylic acid metabolism | 2 | map01210 | Citrate;L-tryptophan; Phenylpyruvic acid |  |
| Phenylalanine metabolism | 2 | map00360 | Phenylpyruvic acid; N-acetyl-l-phenylalanine |  |
| Purine metabolism | 2 | map00230 | Sulfuric acid; 33517 |  |
| Metabolic pathways | 19 | map01100 | L-2-succinylamino-6-oxoheptanedioicacid; Sulfuricacid; Citrate; Sucrose; Phosphoricacid; L-gulonolactone; L-serine o-phosphate; 33517; Vanillyl mandelicacid; L-dopa; N-formylkynurenine; Tryptamine; L-tryptophan; Kynurenicacid; Phenylpyruvic acid; 5-hydroxyindole-3-aceticacid; Indole-3-acetic acid; Indole-3-pyruvic acid; Homovanillic acid |  |

**Supplemental Table S3** The predicted characteristics of selected promoters

| Promoter | Controlling gene | Gene function | -10 region | -35 region | RBS region |
| --- | --- | --- | --- | --- | --- |
| *P_23145_* | *PPSC2_23145* | copper amine oxidase N-terminal | CATAAT | ATGACT | AGGAGG |
| *P_15240_* | *PPSC2_15240 / PPSC2_15235 / PPSC2_15245* | formate C-acetyltransferase / pyruvate formate lyase-activating protein / bifunctional acetaldehyde-CoA / alcohol dehydrogenase | TATCAT | TTTATG | GGAGGG |
| *P_22955_* | *PPSC2_22955* | ribosome-associated translation inhibitor RaiA | TGTAAT | TTGCAG | AGGAGG |
| *P_13385_* | *PPSC2_13385* | hypothetical protein | TATTTT | TTTACG | AGGAGA |
| *P_04420_* | *PPSC2_04420* | DoxX family membrane protein | TAGAAT | TTGATT | GAGGAG |
| *P_05390_* | *PPSC2_05390* | NHLP leader peptide family natural product precursor | TATAAT | TTGCAA | GGAGGA |
| *P_09115_* | *PPSC2_09115* | hypothetical protein | TATAAT | TTGCAT | AGGAGG |
| *P_22445_* | *PPSC2_22445* | elongation factor Tu | AAAAAT | TTTCCA | AGGAGG |
| *P_21510_* | *PPSC2_21510 / PPSC2_21515* | cytochrome ubiquinol oxidase subunit IV / cytochrome ubiquinol oxidase subunit III | TATGAT | TTGTAT | AAGGGG |
| *P_23030_* | *PPSC2_23030* | Flagellin | TAAAAT | CGGAAA | GGAGGA |
| *P_10125_* | *PPSC2_10125* | 30S ribosomal protein S1530S | TAGCTT | TTGTGT | AGGAGG |
| *P_22340_* | *PPSC2_22340 / PPSC2_22335* | 50S ribosomal protein L1550S L15 / preprotein translocase subunit SecY | TCTACT | TTACCG | AGGGAG |
| *P_15565_* | *PPSC2_15565* | HPr family phosphocarrier protein HPr | TATCAT | TGGATG | GGAGAA |
| *P_00160_* | *PPSC2_00160* | Veg protein | TATAAT | TTGACA | AAGAGG |
| *P_25430_* | *PPSC2_25430* | single-stranded DNA-binding protein | TAAAAT | ATGCAA | AGGAGG |
| *P_22450_* | *PPSC2_22450* | elongation factor G | TATTCT | TTTTTG | GGGTGG |
| *P_16895_* | *PPSC2_16895* | GatB / YqeY domain-containing protein | TATAAT | TTGACA | AGGGAG |
| *P_22370_* | *PPSC2_22370* | type Z 30S ribosomal protein S14 | TAAGAT | TTGCAG | GGGTGG |
| *P_00970_* | *PPSC2_00970* | phosphopyruvate hydratase | TAAATT | TTGATC | GGAGGA |
| *P_19355_* | *PPSC2_19355* | ribosomal-processing cysteine protease Prp | TATAAT | TTGAAT | AGGAGG |
| *P_22425_* | *PPSC2_22425* | 50S ribosomal protein L23 | TAATAT | ATGACA | AGGAGG |
| *P_spo0A_* | *PPSC2_15030* | sporulation transcription factor spo0A | AAAAAT | TTGAAT | AGGAGG |

**Table S4** The sequence characteristics of original *P_04420_* and its modified derivatives

| Promoter | Promoter Length | -35 Box | Spacer | -10 Box | | Putative RBS | |
| --- | --- | --- | --- | --- | --- | --- | --- |
|  |  |  |  | sequence | Distance from start codon | Sequence | Distance from start codon |
| P_04420_ | 262 | TTGATT | 14 | TAGAAT | 170 | GAGGAG | 14 |
| P_04420-1_ | 233 | TTGATT | 14 | TAGAAT | 170 | GAGGAG | 14 |
| P_04420-2_ | 205 | TTGATT | 14 | TAGAAT | 170 | GAGGAG | 14 |
| P_04420-3_ | 259 | TTGACA | 14 | TATAAT | 170 | GAGGAG | 14 |
| P_04420-4_ | 266 | TTGATT | 18 | TAGAAT | 170 | GAGGAG | 14 |
| P_04420-5_ | 127 | TTGATT | 14 | TAGAAT | 35 | GAGGAG | 14 |
| P_04420-6_ | 264 | TTGATT | 14 | TAGAAT | 172 | TAAGGAGG | 14 |
| P_04420-7_ | 262 | TTGATT | 14 | TAGAAT | 170 | GAGGAG | 14 |
| P_04420-8_ | 257 | TTGATT | 14 | TAGAAT | 165 | GAGGAG | 9 |
| P_04420-9_ | 254 | TTGATT | 14 | TAGAAT | 162 | GAGGAG | 6 |
| P_n-04420_ | 256 | TTGATT | 14 | TAGAAT | 164 | GAGGAG | 8 |


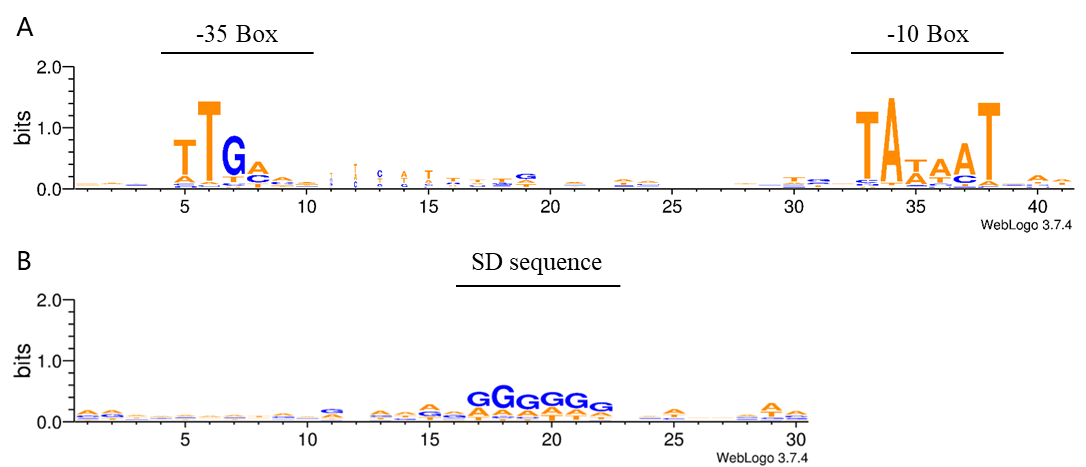


**Figure S1** Schematic representation of conserved bases (-10, -35, and SD regions) in the promoter sequences of 77 high transcription level genes.

**
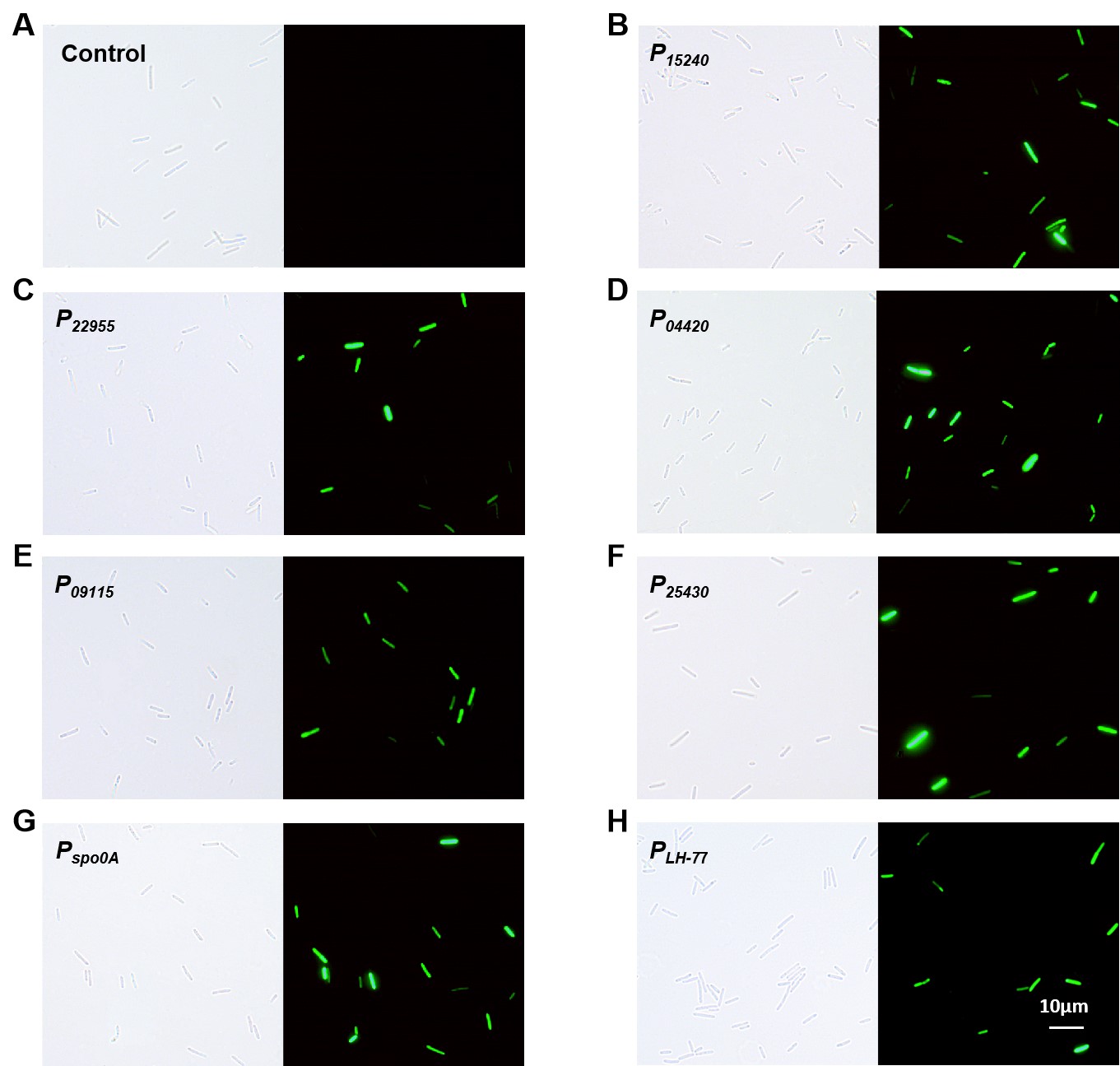
**

**Figure S2** Fluorescent microscopic observation of GFP that expressed by high strengh promoters in strain SC2-M1. The growth time of all recombinant bacteria was 24 h, the laser intensity was 25, the exposure value was 85, and the gain value was 100. The left side of each picture was captured in bright field, and the right side was captured in the corresponding dark field when the blue excitation light was turned on. A was the negative control with no promoter, B to G were the experimental group with studied promoters *P_15240_*, *P_22955_*, *P_04420_*, *P_09115_*, *P_25430_*, and *P_spo0A_*, respectively, and H was the positive control with promoter *P_LH-77_*.

**Figure S3** Fluorescence intensity of GFP that expressed by different promoters. Fluorescence intensity of GFP in *E. coli* DH5α (A) and *B. subtilis* 168 (B). Control represented the *E. coli* DH5α and *B. subtilis* 168 without promoter. The others represented the *E. coli* DH5α and *B. subtilis* 168 with different promoters.

**B**

**A**


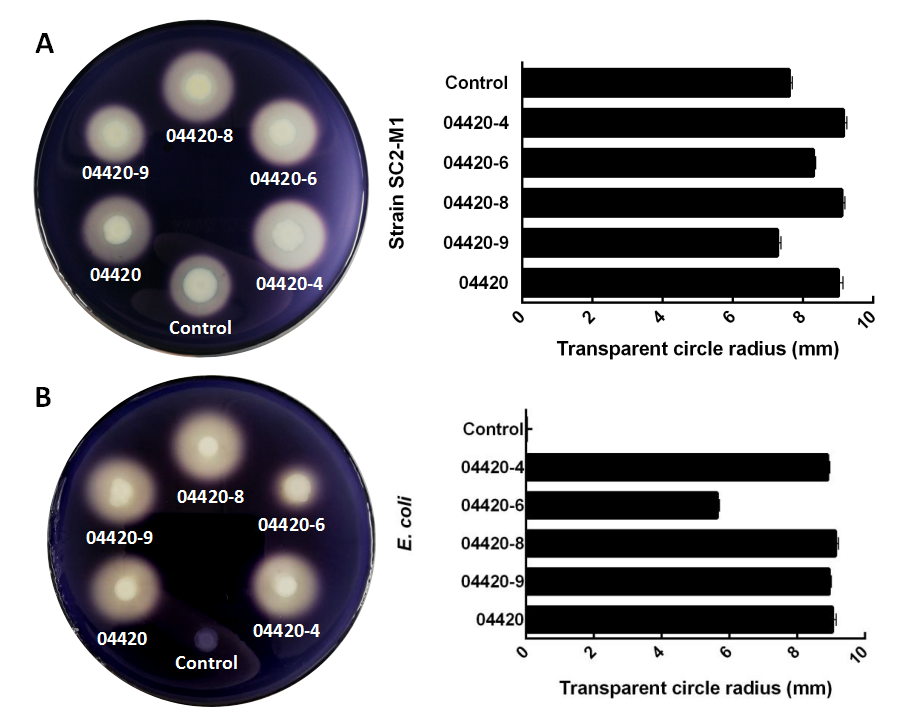


**Figure S4** Assay of α-amylase activities by starch hydrolysis on plates. Recombinant bacteria were grown for 48 hours and the transparent circle radius were observed and caculated. Recombinants of *P. polymyxa* SC2-M1 (A) and *E. coli* DH5α (B). The control contained the empty plasmid pHY300PLK. Promoters *P_04420-4_*, *P_04420-6_*, *P_04420-8_*, *P_04420-9_*, and *P_04420_* were represented with different express activities.
